# The oral microbiome in alcohol use disorder: a longitudinal analysis during inpatient treatment

**DOI:** 10.1080/20002297.2021.2004790

**Published:** 2021-12-01

**Authors:** JJ Barb, KA Maki, N Kazmi, BK Meeks, M Krumlauf, RT Tuason, AT Brooks, NJ Ames, D Goldman, GR Wallen

**Affiliations:** a Translational Biobehavioral and Health Disparities Branch, National Institutes of Health, Clinical Center, Bethesda, MD, USA; b Center for Scientific Review, National Institutes of Health, Bethesda, Md, USA;; c National Institutes of Health, Clinical Center, Bethesda, MD, USA; d Office of the Clinical Director, Laboratory of Neurogenetics, National Institute on Alcohol Abuse and Alcoholism, National Institutes of Health, Bethesda, Md, USA

**Keywords:** Oral microbiome, alcohol use disorder, ion torrent, 16s rRNA, boas, periodontal disease, hmp

## Abstract

**Background:**

Alcohol use disorder (AUD)-induced disruption of oral microbiota can lead to poor oral health; there have been no studies published examining the longitudinal effects of alcohol use cessation on the oral microbiome.

**Aim:**

To investigate the oral microbiome during alcohol cessation during inpatient treatment for AUD.

**Methods:**

Up to 10 oral tongue brushings were collected from 22 AUD patients during inpatient treatment at the National Institutes of Health. Alcohol use history, smoking, and periodontal disease status were measured. Oral microbiome samples were sequenced using *16S rRNA* gene sequencing.

**Results:**

Alpha diversity decreased linearly during treatment across the entire cohort (*P = *0.002). Alcohol preference was associated with changes in both alpha and beta diversity measures. Characteristic tongue dorsum genera from the Human Microbiome Project such as *Streptococcus, Prevotella, Veillonella* and *Haemophilus* were highly correlated in AUD. Oral health-associated genera that changed longitudinally during abstinence included *Actinomyces, Capnocytophaga, Fusobacterium, Neisseria* and *Prevotella*.

**Conclusion:**

The oral microbiome in AUD is affected by alcohol preference. Patients with AUD often have poor oral health but abstinence and attention to oral care improve dysbiosis, decreasing microbiome diversity and periodontal disease-associated genera while improving acute oral health.

## Introduction

Alcohol use disorder (AUD) is characterized by impaired restraint and decision-making leading to excessive alcohol consumption, despite serious health and personal consequences. The prevalence of AUD is rapidly growing in the United States and alcohol abuse-related costs reached $249 billion dollars in 2010 [1]. More than a quarter of adults in the United States reported binge drinking [2,3], and approximately one-third met the Diagnostic and Statistical Manual of Mental Disorders-5 (DSM-5) criteria for AUD at some point in their lives [3]. In AUD, increasing alcohol consumption is accompanied and driven by increased tolerance, craving and withdrawal, and often leads to impairment of executive function and negative emotionality. Patients may abuse alcohol for many years before diagnosis and treatment [2,3], and often neglect their health leading indirectly to a variety of diseases and to late diagnosis of disease. Physiologic effects directly associated with heavy alcohol use contribute to the exacerbation or development of chronic health conditions through alcohol’s damaging effects on most organs and tissues of the body [4]. AUD has been tied to increased risk of several disorders that are themselves associated with severe periodontal disease, including cardiovascular disease, diabetes and oral cancer [5,6].

Periodontal disease and periodontitis are the infiltration and infection of the subgingival tissue by pathogenic bacteria [7]. Periodontitis is more common in patients with AUD due to sustained alterations to the oral ecosystem that occur with continuous alcohol consumption [8]. Alcohol abuse is often associated with neglect of preventative oral care, and disruption of the oral environment by reducing saliva production, decreasing oral pH, increasing the rate of biofilm production and disrupting tooth enamel resulting in cavities and periodontal inflammation [9]. In a cross-sectional study investigating the oral health of patients with AUD versus controls, there were more dental caries, periodontitis, mucosal lesions and decayed, missing and filled permanent teeth (DMFT) values observed [10]. The balance between benign versus dysbiotic microorganisms is emerging as an important risk factor for a variety of diseases including periodontitis associated with AUD [11]. The Human Microbiome Project (HMP1, 2008–2013) provides a large characterization of 16S rRNA sequencing data of the oral microbiome from healthy subjects [12,13] and can be interrogated for comparisons between healthy individuals and individuals with illness.

The oral microbiome is the assemblage of microorganisms that inhabit the oral cavity [14] while transiently or durably occupying different habitats of the mouth [15]. The oral microbiome is a complex ecosystem, with more than 700 taxa of bacterial, fungal and other organisms. As compared to the gut microbiome, oral health and homeostasis are defined by colonization of a smaller number of spatially organized core microbial species [16,17]. Mucosal and non-mucosal surfaces of the tongue dorsum, teeth and gingiva provide unique environments that can be sampled to interrogate different bacterial populations [18]. Changes in the oral microbiome have been associated with periodontitis and oral cancer [19], but systemic diseases, including hypertension and obesity [20], also have links with the oral microbiome [21,22]. There is a growing body of literature detailing interactions between alcohol abuse and the gut microbiome [23–26], but research on the impact of sustained heavy alcohol use and AUD on the oral microbiome is limited [23]. Additionally, it is unknown if oral dysbiosis associated with AUD can be reversed with alcohol use cessation through AUD treatment-associated abstinence.

The aim of this analysis was to explore the influence of alcohol consumption and type, and to longitudinally follow temporal change in the oral microbiome in newly abstinent individuals undergoing inpatient treatment for AUD. Furthermore, two secondary aims of this work were to compare the abundances of health- and periodontitis-associated previously identified genera to those in individuals with AUD and to compare highly abundant oral taxa from healthy individuals to those from individuals with AUD.

## Materials and methods

### Study participants

Twenty-three individuals with AUD were enrolled in a clinical research protocol approved by the National Institutes of Health (NIH) Institutional Review Board (IRB), (NCT02911077). Eligibility was established and clinical assessment and treatment were delivered under an NIH intramural IRB-approved natural history protocol (NCT02231840). The study was conducted over the course of one year (September 2016–August 2017). A total of 53 participants were screened and 23 met eligibility requirements. All individuals participating in the study were able to provide consent. One individual left the NIH Clinical Center (CC) patient care unit within 12 hours of admission, resulting in 22 subjects included in the final analysis. Four individuals did not complete the entire specimen collection schedule but were included. Inclusion criteria were as follows: met DSM-IV [27] or DSM-5 [28] criteria for AUD, able to provide consent, admitted to the NIH CC for inpatient detoxification treatment, ≥ 18 years of age, and body mass index < 30 kg/m^2^. Individuals were excluded from the current study if they had declared taking any of the following drugs by self-report: antibiotics, corticosteroids, immunosuppressive or cytotoxic agents within the last month. Individuals taking experimental medication or enrolled in a research study that could potentially affect the microbiome were excluded. Additionally, individuals who had taken probiotics or who had surgery of the gastrointestinal tract in the past 5 years were excluded.

### Alcohol use and smoking history collection

Alcohol use history and smoking status were collected via the natural history protocol. The Alcohol Timeline Followback (TLFB) and Lifetime Drinking History (LDH) were collected at baseline during the first week after admission [29]. Main outcomes of the TLFB were average drinks consumed per day, number of drinking days, number of heavy drinking days and total number of drinks over the period of last 90 days prior to admission. Average drinks per day recorded via the TLFB were used to categorize the patients into: ‘less-heavy drinkers’ (LHD) or ‘very-heavy drinkers’ (VHD) based on whether they consumed 10 or more drinks per day on average in the 90 days prior to admission, in line with previous methods [23]. The preferred type of alcoholic beverage consumed [‘alcohol choice’] was extracted from the medical history at admission. Alcohol choices were categorized as: ‘beer’, ‘wine’, ‘beer and liquor’ or ‘liquor’. Patients in the beer and liquor category drank beer plus a variety of other liquor beverages including: tequila, gin, whiskey, vodka and liqueurs. Smoking status was recorded using the smoking history questionnaire collected during the first week. Responses were coded as ‘yes’ only for current smokers. Other clinical data were collected as part of the research protocol and were previously outlined in Ames et al. [23] but were not relevant to this oral microbiome analysis and are not reiterated here.

### Oral assessment and specimen collection

Tongue brushings were collected daily during the first week and then weekly for the remaining three weeks for a total of up to 10 samples (Supplemental Figure 1). Patients were asked not to eat, drink or perform oral care for 2 hours prior to collection (small sips of water were permitted). The tongue dorsum was brushed from front to back with a sterile cytology brush for 10–20 seconds. Each brushing was placed into an Eppendorf tube with 1 mL phosphate-buffered saline, placed into a refrigerator and processed within 8 hours. Each sample was vortexed, and then centrifuged at 13,000 rpm for 5 minutes. The pellets were stored at −80°C until DNA extraction. At each oral microbiome collection time point, the modified Beck’s Oral Assessment Scale (BOAS) was collected by nurses who were members of the research team to evaluate acute oral health at sample collection [30]. The modified scale consists of five subscales (assessment of lips, oral mucosa, tongue, teeth and saliva). High BOAS scores indicate poor oral health with the overall possible range to be 5 (no oral dysfunction) to 20 (severe oral dysfunction). The National Institute of Dental and Craniofacial Research (NIDCR) dental team at the Clinical Center performed a complete oral examination for each patient during the first week of inpatient admission. Two oral health assessments were performed: DMFT scoring and periodontal health assessment. Patients were diagnosed with none, mild, moderate or severe periodontal disease based on clinical attachment loss and periodontal pocket depth. For this analysis, patients were further recoded into one of two periodontal disease status groups: (1) none and mild (N/M), and (2) moderate and severe (M/S).

### DNA preparation and 16S rRNA hypervariable region amplification

DNA preparation and amplification of *16S ribosomal ribonucleic acid (rRNA)* gene regions are detailed in Ames et al. (2020) [23]. Briefly, seven hypervariable (V) regions of the *16S rRNA* gene (V2, V3, V4, V6-7, V8, V9) were amplified using the Ion 16S™ Metagenomics Kit (ThermoFisher Scientific, Waltham, MA). Data from all V regions were included in this analysis except that from V9 based on previous findings [31]. Amplified DNA was pooled, and purified, and barcoded libraries were created for each sample using the Ion Fragment Kit (ThermoFisher Scientific, Waltham, MA). Purified and quantitated libraries were diluted to 100 pM and templated to beads using the Ion PGM HiQ View One Touch 2 System and the One Touch 2 520/530 kit (ThermoFisher Scientific, Waltham, MA). Templated libraries were sequenced on the Ion Torrent S5 XL semiconductor sequencer with an Ion 530 chip. Four samples were re-sequenced because they did not meet a threshold of 150,000 reads, making a total of 216 fastq files submitted for bioinformatics processing. Each chip included mock microbiome samples as quality control standards: ATCC® MSA-1002 and ATCC® MSA-1003 (American Type Culture Collection, Manassas, VA).

### Processing of sequences

For more detailed information about sequencing processing, see **Supplemental Methods**. Briefly, bacterial DNA sequences were mapped with Ion Torrent Suite Version 5.0.5. Data were preprocessed using a method described previously [23]. In the USEARCH pipeline for zero-radius operational taxonomic units (ZOTUs) generation, singleton ZOTUs were removed from filtered and trimmed sequences using default parameters in the fastx_uniques command, denoised reads were generated using UNIOISE3, and a ZOTU table for each V region was created using the otutab command [32]. Both ZOTU and taxonomy tables for each V region were exported and imported into the JMP™ Data Discovery software (SAS Headquarters, Cary, NC) for data merging and manipulation.

ZOTU’s were summarized at the genus level by summing ZOTU’s mapped to the same genus for each sample. Each genus level table for each V region were merged by genus using our previously introduced data combination method [23]. Count data for each V region were combined into ‘reconstructed counts’ using the root mean square where x_1_ through x_5_ refers to summed genus counts for V2, V3, V4, V6-7 and V8, respectively:

$$RMS=\sqrt{\frac{{x_{1}}^{2}+{x_{2}}^{2}{x_{3}}^{2}{x_{4}}^{2}{x_{5}}^{2}}{5}}$$

Reconstructed counts were converted to relative abundances (RA) at the genus level for all V regions.

### Statistical analyses

Statistical analyses were performed using JMP™ version 14 Statistical Discovery Software (SAS Headquarters, Cary, NC). See Supplemental Methods for details on the statistical tests used.

Composition of individual oral samples was evaluated by the Shannon diversity index (SDI) and the total number of genera to measure richness and evenness of individual bacterial communities. The total number of genera in each sample was calculated by summing up the total genera with a count of 2 or greater.

To evaluate the compositional difference between clinical variables of interest (VHD/LHD groups, alcohol choice, smoking status and periodontal disease) at each sample and in the cohort over time (sample two versus three-ten), beta diversity was calculated using a Bray-Curtis dissimilarity matrix, and relationships between baseline and longitudinal samples during inpatient treatment were tested using analysis of similarities (ANOSIM). Binary Sorenson-Dice Dissimilarity (BSDD) Index was also used to assess the microbiota dissimilarity between each patient’s baseline sample (first sample collected) compared to each successive sample thereafter, (days 2–7 and weeks 2–4) thus, up to nine samples during treatment [33]. This dissimilarity measure summarizes the overlap of genera found to be present between two samples.

### Genera comparison between tongue dorsum samples from the Human Microbiome Project and individuals with AUD

Tongue dorsum samples from individuals with AUD were compared to tongue dorsum samples from healthy individuals from the Human Microbiome Project (HMP) [12]. The aim of this comparison was to investigate how genera found to be highly abundant from tongue dorsum samples in healthy individuals from the HMP cohort compared to genera found in oral microbiome samples of individuals with AUD. This was an exploratory analysis used to gain a broader understanding if highly abundant genera are highly affected by chronic alcohol use. Since the study design did not include healthy control samples, this analysis was included as a secondary comparison between healthy individuals and those with AUD.

For comparison of our AUD cohort to HMP controls, average relative abundance (RA) values of highly abundant genera in HMP were compared to our AUD cases using Pearson correlation. The level of agreement for each genus was assessed using the residuals generated from the line of identity. Any residual with a 0 indicated perfect agreement between the two genera compared and residuals with increased absolute values indicated increasing discordance between the genera.

### Characterization of ‘health-associated’ and ‘periodontitis-associated’ genera

Genera previously reported by Wilbert et al. to be abundant on the tongue dorsum of healthy individuals was investigated in this patient cohort with AUD [34]. To quantify bacterial taxa (genus-level) that are specific to the healthy human tongue dorsum, we used a two-part validation: (1) taxonomy tables from the HMP were used to identify taxa on healthy tongue dorsum samples and (2) taxa from tongue dorsum samples identified in previously published research. For more detailed methods, please see **Supplemental Methods** for a list of the genera interrogated. Additionally, genera found in shallow and deep sites of plaque samples in periodontal disease from previous research, deemed ‘periodontitis-associated’, were investigated in this cohort of individuals with AUD [35,36].

### Longitudinal relative abundance changes of genera during treatment for AUD

The longitudinal relative abundance change for each genus was investigated between multiple time points during treatment. Each genus in the dataset was tested using a Wilcoxon signed-rank test for paired data between multiple time point comparisons as follows: day 2 versus day 7, day 2 versus week 2, day 2 versus week 3 and day 2 versus week 4. See Supplemental Methods for more detailed methodology. The average RA of the combined ‘health-‘ and ‘periodontitis-‘ associated genera in the HMP were plotted against the same genera in our cohort at day 2 and again at week 4. We determined overall agreement between the RA of ‘health-‘ and ‘periodontitis-‘ associated genera in HMP and AUD individuals by Pearson correlations at day 2 and week 4.

To assess differences between the periodontal disease groups (N/M versus M/S) in this patient cohort and in the periodontitis-associated genera, the two periodontal disease groups were compared in the baseline samples (average of days 1 and 2 samples) and in the end of treatment samples (average of the weeks 3 and 4 samples) using a Wilcoxon signed-rank test.

## Results

### Patient demographics and other clinical measures

All patients met the criteria for Diagnostic and Statistical Manual of Mental Disorders-IV or −5 Moderate or Severe AUD (Table 1). The average age of our cohort was 45.82 ± 13 years, 14 (64%) were male, 14 (64%) were categorized into the VHD group (≥ 10 drinks/day), 8 (36%) into the LHD group (< 10 drinks/day), average BMI was 23.9 ± 2.5 and there were 16 (72%) smokers.

**Table 1.** Participant demographics

**Demographics**	Total Cohort *n* = 22
**Age**	
Mean ± SD	45.82 ± 13.0
**Sex**	***n*** (%)
Male	14 (63.6%)
**Race**	***n*** (%)
White	13 (59.1%)
Black	6 (27.3%)
Multiple	2 (9.1%)
Unknown	1 (4.5%)
**Body mass index**	
Mean ± SD	23.87 ± 2.55
Minimum and maximum	19.0–29.0
**Smoking**	***n*** (%)
Yes	16 (72.72%)
Pack years (*n* = 14)	
Mean ± SD	21.68 ± 16.61
Minimum and maximum	0.50–60.0
**Periodontal assessment**	***n* (%)**
No periodontitis	3 (13.6%)
Mild periodontitis	2 (9.1%)
Moderate periodontitis	14 (63.6%)
Severe periodontitis	3 (13.6%)
**Decayed missing filled teeth (DMFT)**	
mean± SD	10.1 ± 5.9
Minimum and maximum	2–28
**Becks oral assessment score (BOAS)**	
Mean± SD	6.16 ± 1.88
Minimum and maximum	5–13
**Alcohol choice**	***n* (%)**
Beer	4 (18.2%)
Wine	7 (31.8%)
Liquor	6 (27.3%)
Beer/liquor	5 (22.7%)
**Alcohol dependence scale**	N = 21
Mean ± SD	21.1 ± 6.07
Minimum and maximum	11–36
**Timeline followback** (90 days) N = 21* Drinking days Mean ± SD Median Minimum and maximum Average drinks/day Mean ± SD (90 days) Median Minimum and maximum Total drinks Mean ± SD Median Minimum and maximum Heavy drinking days Mean± SD Median Minimum and maximum	81.38 ± 15.21 88.0 29–90 16.20 ± 10.59 13.54 3.3–40 1457.56 ± 952.82 1219 299.3–3600 76.57 ± 22.51 85.00 3–90

*One participant refused to fill out the TLFB questionnaire and therefore the aggregate statistics for this section refer to an *n* = 21.

### Oral health in Alcohol Use Disorder

The cohort had a mean DMFT of 10.1 ± 5.9 when admitted for treatment and was comprised of 19 (86%) individuals with some form of periodontal disease; 17 of those 19 (77%) had moderate to severe periodontal disease. There was no association between DMFT values and the level of alcohol consumption (ANOVA, F_1, 20_: 3.8, *P*= 0.06); however patients with moderate and severe periodontal disease had higher DMFT scores (11.35 ± 5.8) than patients with no or mild periodontal disease (5.8 ± 4.4) but this difference was not significant (ANOVA, *P*< 0.06) (Supplemental Figure 2(a)). On admission, the average BOAS score across all individuals was 7.4 ± 2.9, and during treatment, the BOAS decreased significantly over time across all patients, indicating improved oral health (Linear fixed effect model, F_1, 21.49_: = 4.7, *P = *0.04; Supplemental Figure 2(b)). When assessing BOAS scores on admission between the periodontal disease groups, the N/M group had a lower mean BOAS (5.4 ± 0.54) compared to the M/S group (8.0 ± 3.04); however, this difference was not significant (ANOVA, *P* < 0.08).

### Sequencing details

We analyzed up to 10 oral microbiome samples in each of the 22 individuals with AUD over the course of 28 days resulting in 212 total oral samples. Oral microbial community structure was assessed using *16S rRNA* gene amplicon sequencing at regions V2, V3, V4, V6-7 and V8. In total, 212 FASTQ files, including 78,289,538 sequence reads were analyzed with an average of 362,451 ± 225,994 sequences per sample. The average read length across the samples was 247 base pairs. A failed sample (18 samples in total) and a sample that did not undergo proper storage were removed from further processing and analysis (two samples in total). Sequence depth was rarefied to 5000 sequences per sample for calculating alpha diversity indices. Data analyses were performed at the taxonomic level of genus. Once data from the six V regions were combined into reconstructed counts at the genus level, a total of 144 genera and 210 samples (after removal of low read samples) were submitted for downstream analysis.

### Oral microbial diversity in AUD during treatment

**Figure 1.**
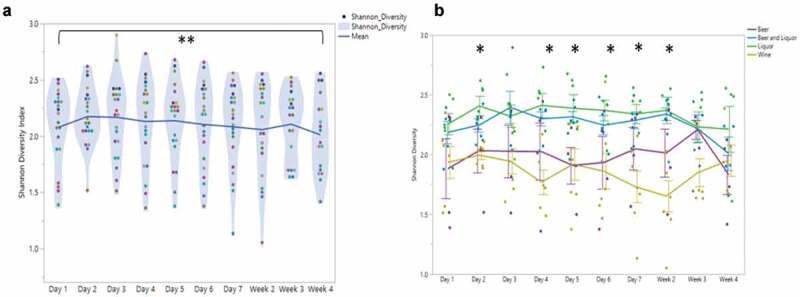
Alcohol Use Disorder oral microbial diversity during treatment

**Figure 2.**
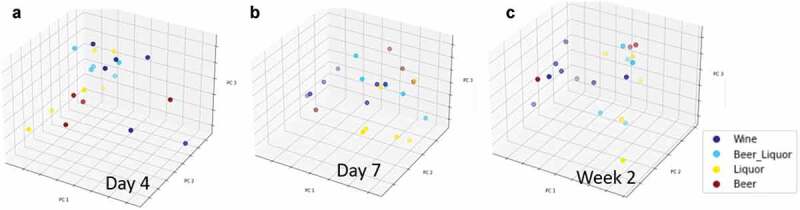
Beta diversity differences by alcohol of choice groups

There was a significant decrease in microbial diversity (SDI) at week 4 (2.01 ± 0.34) compared to SDI at day 2 (2.17 ± 0.25); (Matched Pairs *T*-test, *t* = −3.11, *P = *0.006) (Figure 1(a)). SDI linearly decreased across the 4 weeks of inpatient treatment, (Linear fixed effect model, F_1, 18.85_ = 13.1, *P = *0.002). Differences in average SDI were assessed at each sampling time point stratified by four variables of interest: drinking consumption (VHD, LHD; Supplemental Figure 3(a)), periodontal disease status (N/M, M/S; Supplemental Figure 3(b)), smoking status (yes, no; Supplemental Figure 3(c)) and alcohol choice (beer, beer and liquor, liquor, wine; Figure 2(b)). Alcohol consumption, smoking and periodontal disease were not associated with SDI at any time point, however, at day 1 smokers had a lower SDI average than non-smokers (ANOVA or Student’s *T*-test, *t* ratio: −2.08, *P*= 0.05; Supplemental Figure 3(d)). There was no significant association between alcohol choice groups and time throughout treatment (Repeated Measures ANOVA, group × time F_27, 152.1_ = 1.25, *P = *0.20), but alcohol choice had a significant main effect on SDI (group F_3, 17.97_ = 5.35, *P = *0.008). Significant SDI differences between alcohol choice groups was observed at day 2, days 4–7 and week 2 (ANOVA, *P* = 0.007, *P = *0.006, *P = *0.02, *P = *0.04, *P = *0.004 and *P = *0.002, respectively; Figure 1(b)). Throughout the course of inpatient treatment, wine drinkers had the lowest SDI (yellow), compared to beer (purple), liquor (green) and beer and liquor drinkers (blue). Beer only drinkers had the second-lowest average SDI over all oral microbiome samples (1.98 ± 35). No significant association between SDI and periodontal disease (Repeated Measures ANOVA, group × time F_9, 170_ = 0.48, *P = *0.88), smoking status (Repeated Measures ANOVA, group × time F_9, 170_ = 1.34, *P = *0.22), or drinking consumption groups (Repeated Measures ANOVA, group × time F_9, 170_ = 0.7, *P = *0.71), was found over time. The total number of genera were significantly different across the alcohol choice groups at days 1 (ANOVA tests at each time point) (*P = *0.03), 4 (*P = *0.02) and 7 (*P = *0.02) and at weeks 2 and 4 (*P = *0.03, *P = *0.04, respectively; Supplemental Figure 4).

**1A**) Average Shannon Diversity Index (SDI) at each sampling time point through treatment. Each point represents an individual’s SDI at that sampling time. Each color represents the same patient through treatment. Center horizontal line represents the average SDI across each sampling time point. Comparison between SDI at day 2 and week 4 is significantly different, noted by double asterisk (*P*< .006).**1B**) Shannon Diversity index (y-axis) at each sampling time point (x-axis) by alcohol choice. Beer (purple) and wine drinkers (yellow) had lower average SDI during treatment than did beer and liquor (blue) and liquor drinkers (green). Significant SDI changes between alcohol preference types were observed at days 2, 4–7 and week 2. Repeated measures mixed model analysis reported significant main effect of alcohol preference (F: 5.3, *P < *.008).

**A–C**. Principal Components Analysis (PCA) plots of oral microbiome samples, performed at the taxonomic level of genus and based on Bray-Curtis dissimilarity stratified by alcohol of choice groups. Overall gut microbial community structure differences between alcohol of choice groups varied by sample, as determined by analysis of similarity (ANOSIM) analysis, at **A**: day 4 (R = 0.189, *P* = 0.035), **B**: day 7 (R = 0.170, *P* = 0.035) and **C**: week 2 (R = 0.158, *P* = 0.049)

**Figure 3.**
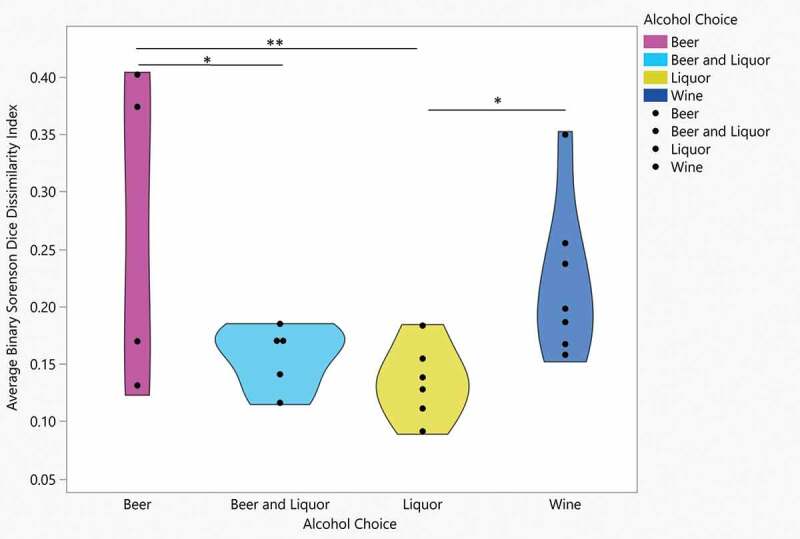
Binary Sorenson Dice Dissimilarity by alcohol choice

Average BSDD across time points for each patient (y-axis) stratified by alcohol choice. Significant difference found over four alcohol choice groups (F:3.6, *P < *0.03). Pairwise significant average BSDD found between Beer and Beer and Liquor (*P < *.03), Beer and Liquor (*P < *.009) and Liquor and Wine (*P < *.04).

**Figure 4.**
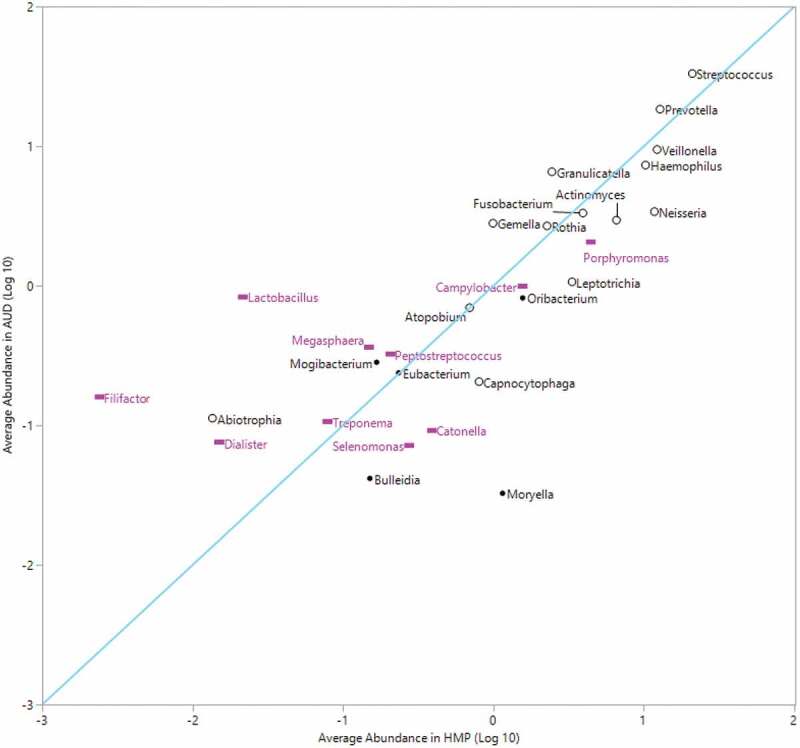
Average relative abundance of top 20% abundant genera in HMP compared to average abundance in AUD

Correlation between the top 20% average abundance HMP genera (x-axis) versus average abundance in AUD (y-axis). Diagonal line represents line of identity. Correlation coefficient (Pearson *r*: 0.75). *N* = 10 filled pink rectangle of periodontitis-associated genera, *N* = 14 black open circle of health-associated genera. (*missing periodontitis-associated genus *Desulfobulbus* in figure. Genus not found in the HMP dataset.)

Although there was a significant linear decrease of oral microbial alpha diversity over 4 weeks of inpatient treatment for AUD, microbial beta diversity analyses did not indicate differences in the observed microbial community structure from day 2 compared to day 3 (ANOSIM based on Bray-Curtis dissimilarity; R = −0.033, *P* = 0.914), day 4 (R = −0.014, *P* = 0.639), day 5 (R = 0.002, *P* = 0.378), day 6 (R = 0.004, *P* = 0.391), day 7 (R = 0.003, *P* = 0.39), week 2 (R = 0.010, *P* = 0.284), week 3 (R = −0.027, *P* = 0.793) or week 4 (R = −0.014, *P* = 0.6; Supplemental Figure 5(a-h)). There were significant microbial community structure differences by alcohol of choice groups at day 4 (ANOSIM based on Bray-Curtis dissimilarity; R = 0.189, *P* = 0.035, Figure 2(a)), day 7 (R = 0.170, *P* = 0.035, Figure 2(b)) and week 2 (R = 0.158, *P* = 0.049, Figure 2(c)). Significant differences in the observed microbial community structure were not seen by VHD/LHD groups (Supplemental Figure 6A-I), smoking status (Supplemental Figure 7A-I) and periodontal disease groups (Supplemental Figures 8A-I), with the exception of smoking status at week 3 (R = 0.223, *P* = 0.021; Supplemental Figure 7 H). Average BSDD values indicating average dissimilarity from baseline for each patient were stratified by drinking group, alcohol of choice, smoking and periodontal disease. Drinking consumption, smoking status and periodontal disease status showed no significant differences in average BSDD, but alcohol choice showed significant average BSDD values across the alcohol choice groups (ANOVA, F_3, 18_ = 3.6, *P = *0.03; Figure 3). When each pair was assessed, average differences were seen between wine and liquor (Student’s *T*-test, *P*= 0.04), beer and liquor groups (Student’s *T*-test, *P*= 0.009), and beer and beer and liquor (Student’s *T*-test, *P*= 0.03).

**Figure 5.**
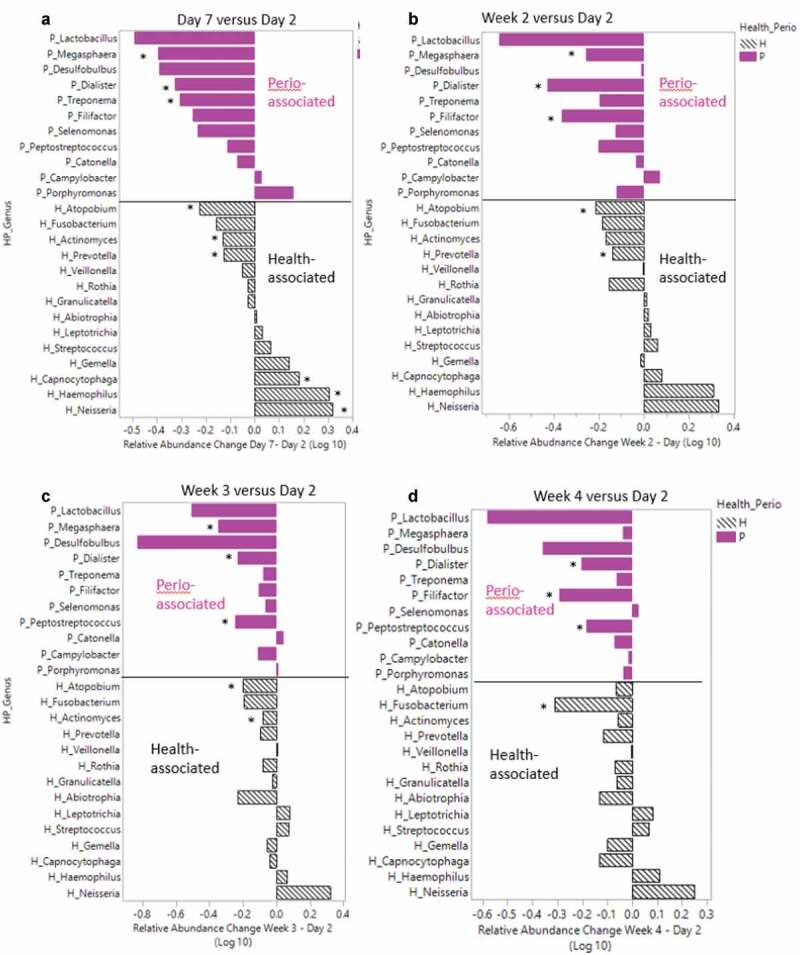
Health and periodontitis-associated genera relative abundance differences between day 2 versus day 7 and weeks 2–4

Average logarithm base 10 relative abundance difference (y-axis) between **5A**) day 7 versus day 2 samples, **5B**) week 2 versus day 2, **5 C**) week 3 versus day 2, and **5D**) week 4 versus day 2. Gray patterned bars represent ‘health’ associated genera and pink solid bars represent ‘periodontal-disease’ associated genera. Asterisk indicates genus that showed significant difference between the associated comparison of interest (day 7 versus day 2, week 2 versus day 2, week 3 versus day 2, week 4 versus day 2). See Supplemental Table 2 for corresponding *p* values.

### Highly abundant genera found on the tongue dorsum in healthy versus AUD

Comparisons between average RA of abundant genera in the HMP tongue dorsum and the individuals with AUD in this study (Figure 4) revealed that the three genera with the highest RA in both datasets were *Streptococcus (*HMP: 21 ± 12%, AUD: 33 ± 14.2%), *Prevotella (*HMP: 13 ± 2.8%, AUD: 18.4 ± 8.8%) and *Veillonella* (HMP: 12.2 ± 6.1%, AUD: 9.4 ± 3.6%). These genera were congruent between HMP and our cohort with residuals of 0.20, 0.16 and 0.11 for *Streptococcus, Prevotella* and *Veillonella*, respectively. However, four highly abundant genera in HMP fell below an average of 0.10% abundance in the individuals with AUD. Those four genera were *Moryella* (average RA: 0.03 ± 0.003%), *Catonella* (average RA: 0.09 ± 0.08%), *Selenomonas* (average RA: 0.07 ± 0.06%) and *Bulleidia* (average RA: 0.04 ± 0.04%). *Filifactor, Lactobacillus* and *Moryella* differed the most between the two sets of data. Despite potential methodological differences, the RA of highly abundant genera HMP displayed strong overall agreement (Pearson Correlation, *r* = 0.82, *P*< 0.0001) with our cohort indicating that the differences that were observed may be biologically meaningful.

### Longitudinal changes of genera during treatment for AUD

Individual patient relative abundances showed inter- and intrapatient variability of specific genera including *Actinomyces* (orange), *Haemophilus* (light blue) and *Streptococcus* (gray) (Supplemental Figure 9). Across all patients, the *Streptococcus* (gray) and *Prevotella* (blue) genera were highly abundant and showed varying relative abundances over the ten sampling time points through treatment. Some patients also showed higher abundances of *Neisseria* (orange) and *Haemophilus* (light blue) than others. To investigate which genera significantly changed longitudinally, relative abundances of day 2 samples were compared using a Wilcoxon signed-rank test comparing day 7 samples and weeks 2–4 samples (Supplemental Table 2 and Supplemental Figure 10). There were 24 genera found to change significantly from admission throughout the course of the treatment program. Of these 23 genera, 13 changed within the first week (day 2 to day 7 comparison). Over the first week, several genera with a significant change over time exhibited a decrease in RA including *Cryptobacterium* (day 2 = 0.018 ± 0.051 versus day 7 = 0.002 ± 0.005, *P* = 0.005), *Parascardovia* (day 2 = 0.011 ± 0.017 versus day 7 = 0.005 ± 0.016, *P* = 0.037) and *Stomatobaculum* (day 2 = 0.560 ± 0.645 versus day 7 = 0.314 ± 0.257, *P* = 0.030). By week 2, 13 genera changed significantly with six genera overlapping from the week 1 comparison (*Atopobium, Cryptobacterium, Dialister, Megasphaera, Parascardovia*, and *Pseudoramibacter*). By week 3, seven genera were found to change when compared to the baseline day 2 sample, with three overlapping between the day 7 and week 2 comparisons (*Atopobium, Cryptobacterium*, and *Dialister*). Finally, by week 4, six genera changed significantly in relative abundance over time. *Dialister* and *Cryptobacterium* changed across all four comparisons (day 2 versus day 7, week 2, week 3 and week 4), and both genera had a consistent decrease in RA from day 2 over time. We quantified average RA changes in health- and periodontitis-associated genera from day 2 to day 7, week 2, week 3 and week 4, respectively, to visualize the impact of alcohol cessation on ‘health-’ and ‘periodontitis-‘ associated genera over time. Over the first week following alcohol cessation (day 2 vs day 7), six healthy and three periodontitis-associated genera changed significantly, with a net increase (0.29 log RA difference) of healthy genera and a net decrease (−2.4 log RA difference) of periodontal disease-associate genera (Figure 5(a)). Of the 11 periodontitis-associated genera, all but two (*Porphyromonas* and *Campylobacter*) decreased in abundance from day 2 to day 7. Some health-associated genera also decreased in RA during the inpatient treatment period for AUD. The RA of *Prevotella* and *Atopobium* was significantly lower at day 7 (Figure 5(a)) and week 2 (Figure 5(b)), compared to day 2, and *Atopobium* was also significantly lower at week 3 (Figure 5(c)), compared to day 2. At week 4, the only genera with a significant decrease was *Fusobacterium*, when compared to day 2 (Figure 5(d)). Abstinence from alcohol during the treatment period was associated with a decrease in periodontitis-associated genera *Filifactor, Megasphaera*, and *Peptostreptococcus. Dialister*, another periodontitis-associated genus, remained significantly decreased from day 2 through the entire course of treatment.

We compared agreement of average RA of health- and periodontitis-associated genera between the HMP and AUD subject cohorts at day 2 and week 4 to evaluate to changes in agreement over time (Supplemental Figure 11). Overall agreement in the average RA of health and periodontitis-associated genera in the HMP and AUD patient datasets improved over time. At day 2, the correlation between the two datasets was 0.62 (Pearson, *P* = 0.0003), and at week 4 agreement increased to a correlation of 0.88 (Pearson, *P* < 0.0001). At day 2, an increased RA of periodontitis-associated genera in the AUD cohort versus HMP and a decreased RA of health-associated genera in the AUD cohort versus HMP categorized the major deviations from the line of agreement. *Filifactor, Lactobacillus* and *Dialister*, all periodontitis-associated genera, had residuals of 1.85, 1.57 and 0.97 at day 2. Conversely, the health-associated genera *Capnocytophaga* and *Neisseria* had residuals of −0.73 and −0.72, respectively. At week 4 the residuals for *Filifactor, Lactobacillus* and *Dialister* improved to 1.23, 0.69 and 0.45, respectively. *Capnocytophaga* residuals improved slightly to −0.65 and *Neisseria* remained consistent at −0.72. An exception to the higher RA of periodontal-associated genera in patients with AUD was *Catonella* with a reported residual of −0.71 at day 2 and −0.81 at week 4.

The relative abundance of periodontal-associated genera were also compared using a Wilcoxon signed rank test by periodontitis disease groups (N/M and M/S) at the start of the treatment (days 1 and 2) and at the end of treatment (weeks 3 and 4; Supplemental Figure 12). The relative abundance of *Lactobacillus (*mean M/S: 1.34 ± 3.93, mean N/M: 0.05 ± 0.11, *P < *.04), *Filifactor (*mean M/S: 0.18 ± 0.38, mean N/M: .01 ± .002, *P < *.04), *Dialister* (mean M/S: .13 ± 0.23, mean N/M: .01, SD: 0.01, *P < *.03) and *Treponema (*mean M/S: 0.15 ± 0.26, mean N/M: 0.01 ± 0.02, *P*< 0.02) were significantly elevated in the M/S periodontal disease group, compared to N/M at the start of treatment (Supplemental Table 3). At the end of treatment, *Dialister* remained significantly higher in the M/S group (mean M/S: 0.05 ± 0.06, mean N/M: 0.01 ± 0.02, *P* < 0.01). At the end of treatment, *Porphyromonas* and *Catonella* were significantly higher in the N/M group, compared to the M/S group (*P*< 0.04 and mean M/S: 0.11 ± 0.1, mean N/M: 0.05 ± 0.07, *P*< 0.03, respectively).

## Discussion

In this exploratory analysis of the oral microbiome in AUD following newly abstinent patients, we present a number of novel findings focused on clinical measures of the oral microbiome of treatment-seeking patients with AUD. A subjective measure of acute oral health, a clinician administered modified Beck’s Oral Assessment Score (BOAS), improved significantly during treatment, while the alpha diversity measured by SDI of the oral microbiota decreased in patients with AUD following alcohol cessation. The amount of alcohol consumed did not appear to be a major predictor of alpha diversity differences, but the preferred alcohol choice contributed to average (alpha and beta) diversity changes during the first two weeks following alcohol cessation. We found that within this patient cohort, smoking and periodontal disease status did not affect the alpha diversity globally, but specific periodontitis associated genera changed during treatment and across differing periodontal disease groups. We also found that individual genera changed during the longitudinal inpatient treatment period when compared to samples at admission (day 2) indicating a significant effect of alcohol cessation of specific oral microbiome taxa. Finally, certain health-associated genera showed the most significant changes during the first week of alcohol use cessation. This works includes a thorough and comprehensive view of the oral microbiome using the Ion 16S Metagenomics Kit (Thermo Fisher Scientific, Waltham, MA) interrogating the hypervariable regions V2, V3, V4, V6-7 and V8. Traditional microbiome studies are designed with one specific primer targeting, at most, one-two V regions of the *16S rRNA* gene thus having the potential to miss certain V region-specific taxa not targeted. This work alleviates this potential bias by incorporating six of the nine V regions of the 16S rRNA into the analysis framework.

The modified BOAS, a clinical measure used for oral health assessment, decreased significantly throughout inpatient treatment for AUD highlighting the notion that alcohol use cessation along with dental health counseling during inpatient treatment can be beneficial to acute oral health, especially given that individuals with heavy alcohol use may not be attentive to good oral health care. A cross-sectional outpatient study in 2017 comparing individuals with AUD to healthy controls showed that individuals with AUD have significantly lower oral hygiene scores [8]. In this study, all patients received a thorough dental examination where the periodontal disease status and DMFT score was recorded by the dental staff. Patients with M/S periodontal disease had a mean DMFT that was 5.55 points higher than the N/M group, although this difference was not statistically significant (*P*= 0.06). The M/S periodontal disease rate in the Average American according to the NHANES survey 1999–2004 was 5% [37]; however, 77% of the patients with AUD in this cohort had moderate to severe periodontal disease. When investigating the BOAS score across the two periodontal disease groups, the M/S group had higher average BOAS scores at the start of the program than the N/M group, but these differences were also not statistically significant. As the treatment program progressed, the average BOAS scores in the M/S periodontal disease group decreased indicating an improvement in oral health in the study cohort during inpatient treatment for AUD. If the BOAS continued to improve over time, we might observe an improvement in periodontal disease status; however, this would need to be confirmed with a prospective follow up dental examination after sustained attention to oral health care. The treatment period in this study was too short to observe an improvement in diagnosed periodontal disease.

Overall, there was a small but significant decrease in oral microbial alpha diversity (SDI) during inpatient treatment following the cessation of alcohol use. When the day 2 sample was compared to the last sample (week 4) during treatment, there was a significant decrease in average SDI by 0.16. Although the oral microbiome is the second-most diverse microbial habitat on the human body (preceded by the gut microbiome) [11], there is a small ‘core’ number of genera/species on the tongue dorsum [34,38]. In studies of the human gut microbiome, decreased alpha diversity is associated with poor gut health [39] and disease [40,41]. As we saw an overall reduction in periodontitis-associated genera over time spent abstinent from alcohol, the reduction in SDI in this patient cohort was likely related to an improvement in oral health. An accumulation of new and/or pathogenic species in the mouth is characteristic of periodontitis, and this would lead to a subsequent increase in oral microbiome alpha diversity compared to periodontitis-free mouths [11]. Other research has also demonstrated relationships between heavy drinking and increased alpha diversity, compared to non-drinkers [42]. Saliva plays a key component in maintaining a neural oral pH around 6.2–7.6, contributing to the growth of many organisms that thrive in healthy mouths [43]. Alcohol is a diuretic, and many patients with AUD are chronically dehydrated [44], contributing to chronic dry mouth and oral mucosal irritation resulting in higher BOAS scores. Given that similar negative slopes in SDI and BOAS were observed during inpatient treatment in this patient cohort, the improvement of acute oral health may be a direct contributing factor to the overall decrease in alpha diversity. Future studies with larger samples sizes in similar longitudinal patient cohorts will be important to support these findings.

We investigated SDI across specific clinical variables such as drinking quantity, smoking, periodontal disease severity and alcohol choice. We did not see differences in SDI over time, when stratifying by drinking consumption (VHD versus LHD) groups. In our previous work assessing gut microbiome samples during inpatient treatment, we reported that the VHD group showed greater global gut microbial changes during treatment compared to the LHD group [23]. Although we did not see a significant impact on smoking on the oral microbiome during the longitudinal sampling, we observed a small reduction in SDI in the reported smokers at admission (day 1 sample), although this difference was not significant (*P* = 0.051). In our study, daily smoking habits during inpatient treatment were not collected and therefore we could not analyze the combined effects of smoking on the longitudinal oral microbiome samples in individuals with AUD. Detailed quantification of tobacco use during abstinence from alcohol in future work will aid in measuring the mediating effects of smoking on the oral microbiome as patients progress through inpatient treatment.

When patients were grouped based on preferred alcohol choice, we observed differences in SDI, Bray Curtis dissimilarity, number of genera and average BSDD indicating a significant influence of alcohol choice on global oral microbiome characteristics. Fan et al. (2018) also evaluated the impact on alcohol choice on the oral microbiome in non-treatment seeking individuals who consumed alcohol enrolled in two large USA cohort studies [42]. Differences in alpha diversity between wine drinkers and non-drinkers were seen in another study of alcohol consumption and the oral microbiome [42], but oral microbiome alpha diversity did not differ significantly between alcohol preference types. Subjects in the study by Fan et al. (2018) who consumed alcohol had significantly lower daily alcohol intake compared to this cohort prior to inpatient admission, which may explain the differences in SDI and genera counts by alcohol preference type throughout the inpatient treatment program. Our research design identified differences in alcohol choice groups across several sampling times and this longitudinal sampling design following abstinence is a strength of our study. SDI differed by alcohol choice during the first two weeks of alcohol abstinence (with the exception of days 1 and 3), while the number of genera identified in the oral microbiome differed at weeks 3–4 in addition to days 1, 3 and 7. Wine drinkers had both an overall lower SDI and number of genera, while liquor and beer and liquor drinkers had higher SDI and/or genera identified throughout the treatment program. Given that an individual consuming beer may consume more volume (12 ounces of volume in a regular beer) of the beverage versus an individual consuming liquor (1.5 ounces in a standard drink), there is likely an association with the oral microbiota and the amount of carbohydrate or sugar density ingested between the differing alcohol groups. These preliminary findings suggest an association between alcohol choice and consumption and a potential mediation of oral dysbiosis changes in oral health over time.

The Bray Curtis dissimilarity measures the overall compositional differences between samples and was used in addition to the BSDD to quantify global microbiome differences between metadata groups. The ANOSIM results showed significant beta diversity of oral microbiome differences, stratified by alcohol choice at day 4, day 7 and at week 2 of inpatient treatment. The BSDD measures the overlap of microbiota between two samples and was used as a measure of dissimilarity across the longitudinal samples. Significant differences were observed between average BSDD values across alcohol choice groups with liquor drinkers showing the lowest level of change when compared to beer drinkers. The high SDI and genera number and low overall change (by BSDD) in liquor drinkers suggested that liquor may have the biggest negative impact on overall oral microbiome community. Beer drinkers had a high number of genera and average change (by BSDD) in the mouth, but lower SDI (versus beer and beer and liquor drinkers) throughout the inpatient period. Differences in alpha diversity and BSDD measures across alcohol type may be related to the volume of alcohol consumed to achieve the same effect (grams/volume of alcohol), sugar content differences or byproducts created from the alcohol fermentation processes. For microorganisms to thrive, nutrients essential for growth need to be available in the microbiome environment, including amino acids, proteins, and glycoproteins [45]. Alcohol decreases the pH in the mouth, shifting the oral ecosystem to favor production of different bacteria, and both the alcohol percentage level and the mixers consumed with alcohol (especially in the case of liquor drinkers) may be broken down to various byproducts. Measuring the volume, usual consumption times and mixers, if present in future studies will continue to inform which factors underlie the differences seen in alcohol types we observed in this study.

When genera between HMP subjects and patients with AUD (all time points) were compared (Figure 4), there was a strong correlation in average RA of highly abundant genera associated with a healthy tongue dorsum, i.e. *Streptococcus, Prevotella* and *Veillonella*. The average RA of *Streptococcus* and *Prevotella* was slightly higher in patients with AUD, while *Veillonella* was higher in the HMP group. Two genera that had notable differences between the HMP and AUD datasets were *Desulfobulbus* and *Moryella. Desulfobulbus* was not identified in any of the HMP subjects at the genus level, while several patients in our study cohort had *Desulfobulbus* present in the taxonomy table. Conversely, the RA of *Moryella* was significantly higher in the HMP dataset with an average RA of 1.149% in HMP patients and 0.033% in patients with AUD. *Desulfobulbus* is associated with advanced periodontal disease [46,47], and is likely pathogenic due to its evasion and neutralization of host immune defense mechanisms in the oral cavity [48]. The relationship of *Moryella* to oral health or disease is not as well defined in the literature, but *Moryella* was differentially increased in caries-free children, as compared to caries-active children [49]. Deeper sequencing or quantification of metabolic genes may provide more insight to the clinical significance of reduced *Moryella* in the mouths of patients with AUD.

Of bacteria that was associated with the healthy oral microbiome by Wilbert et al. (2020), 13 of the 14 were categorized as highly abundant in the HMP dataset (≥ 0.10% average RA). Of these genera that were highly abundant in HMP patients, we found five genera to be represented at less than 0.2% average abundance in AUD including *Eikenella, Staphylococcus, Propionibacterium, Treponema* and *Capnocytophaga*. When comparing longitudinal changes of health-associated and disease-associated genera across the treatment period, the greatest number of genera changed in the first week of inpatient treatment. Other notable bacteria associated with periodontal disease that decreased during the inpatient treatment period were *Megasphaera* and *Filifactor. Lactobacillus* also had large mean decreases in average RA, but changes were not statistically significant due to high interindividual variability in changes across patients. *Dialister*, a periodontal disease associated genus, remained significantly decreased from day 2 through the entire course of treatment. Over the course of the treatment period, there were also differences in the RA of health-associated genera, although a majority of the changes in health-associated genera occurred in the first week.

There were significant decreases in health-associated bacteria including *Atopobium, Prevotella, Actinomyces* and *Fusobacterium* during at least one time point over the treatment period. Nevertheless, in all of these genera, the residuals (measure of agreement) between mean RA of HMP and AUD individuals decreased in week 4 compared to day 2, indicating better agreement with the ‘control’ HMP patient population over time. Therefore, health-associated genera may be present in higher than usual levels in individuals with AUD, which may also contribute to oral dysbiosis. It has been postulated that an overabundance of certain healthy genera can lead to oral dysbiosis and these findings could further implicate this [50]. There were also longitudinal decreases and increases in oral microbiome health-associated bacteria in our cohort during the inpatient treatment period that have been shown to be positively or negatively associated with alcohol use, respectively, in the current literature. For example, the RA of *Capnocytophaga* and *Neisseria* increased during the inpatient period in our subjects. In the current literature, relative abundances of *Capnocytophaga, C. ochracea* and *Neisseria* were decreased in the oral microbiota of alcohol users [51,52] and rats treated with alcohol [53] indicating a bacterial shift closer to non-alcohol using controls over time. Furthermore, the RA of *Actinomyces, Fusobacterium* and *Prevotella* significantly decreased over time when study subjects were abstinent from alcohol. The RA of *Actinomyces* and its constituent species *A. graevenitzii* in the oral microbiome was increased in moderate and heavy drinkers in a previous study [42]. The RA of *Fusobacterium* including *F. nucleatum* in oral microbiome samples have been reported in previous research to be both associated with increased RA [42,54] and decreased RA [51,53] in heavy and/or moderate drinking human or animal groups. Similarly, in previous research, the RA of *Prevotella* in heavy to moderate alcohol intake groups was increased [42,51,55] and decreased [9,53,56] in oral microbiome samples. These variations in the current literature may be related to different confounding variables in the study population, different oral microbiome sampling sites, concurrent increases and decreases in bacterial species with many studies quantifying taxa at the genus level, or a combination of several factors. Future confirmatory research studying the oral microbiome in patients with AUD and heavy alcohol use with higher taxonomic resolution or metabolic gene quantification would inform the functional potential of oral bacteria responses to abstinence from alcohol.

The combined decrease in several periodontitis-associated genera and equivocal change in health-associated genera led to an overall decrease in SDI over time. Although the differences look minimal when looking at the overall response in SDI, the dramatic decreases in several periodontitis-associated bacteria indicate that abstinence from alcohol has a positive impact on the oral microbiome and oral health. Finally, the RA of many periodontitis-associated genera was higher in patients with M/S periodontal disease when compared to those in patients with N/M periodontal disease even when investigating samples from the tongue dorsum, which is expected given the periodontal disease diagnosis. In a 2019 study, tongue dorsum samples were used to investigate systemic disease and especially, periodontal disease, validating that this oral sampling niche can be used to investigate the oral microbiome and oral health [57,58]. In our study, interestingly, *Porphyromonas* and *Catonella* were significantly higher in N/M at the end of treatment (*P < *.04 and mean M/S: 0.11 ± 0.1, mean N/M: 0.05 ± 0.07, *P < *.03 respectively), even though they were previously found to be elevated in periodontitis samples.

We acknowledge certain limitations in this exploratory analysis of the oral microbiome in patients with AUD. This work includes a small sample representation of patients with AUD and cannot thoroughly represent the oral microbiome of all individuals with AUD. Nevertheless, we found exciting preliminary evidence on the impact of abstinence from alcohol on the oral microbiome. The differences found between alcohol choice groups should be followed up in a controlled study sample designed specifically to investigate this topic with a larger cohort of patients. Furthermore, given the small sample size and the small samples within each of the metadata variables of interest, we could not apply advanced data modeling for this design. This study provides important preliminary information for which metadata variables should be adequately powered to confirm their influence on oral microbiome community characteristics so that more advanced data modeling can be applied. Although the experimental design did not include oral samples from healthy patients, an investigation between RA from healthy tongue dorsum samples in the HMP cohort versus individuals with AUD was conducted to allow for comparisons between healthy control tongue dorsum samples and our patient population. While this was an exploratory investigation between the HMP and AUD to gain a broad understanding as to how highly abundant genera in healthy people are affected from chronic alcohol use, we acknowledge that this workflow has its own set of limitations. This analysis was done on a subset of taxa at the genus level. There could be inherent bias reflected because the two datasets were constructed from differing sequencing platforms with different V region amplifications (Illumina V1-3 and V3-5 versus Ion Torrent V2, V3, V4, V6-7, V8) and differing bioinformatics processing methods. Additionally, due to the design of our sequencing kit covering multiple V regions, we were not able to annotate bacteria to the species level. Future research including longitudinal samples of control subjects with shotgun metagenomics or single V region sequencing could determine whether the differences in RA between controls and patients with AUD can be replicated, and if temporal changes in oral microbiome bacteria and the genus and species level occurred in control patients to the same extent over time. The investigation of the link between periodontal disease and the oral microbiome was not a primary focus of this study because we collected tongue dorsum samples and did not specifically interrogate plaque samples. A more detailed focus on specific oral sites within the mouth of patients with periodontal disease should be conducted to follow up on our results. More than 77% of this patient cohort had moderate to severe periodontal disease, and a balanced comparison between the M/S and N/M periodontal disease groups including other oral sample sites such as saliva, supragingival and subgingival plaque would inform which oral microbiome changes are related to the periodontal disease and which are more strongly attributed to alcohol consumption. We believe that this study provides a detailed exploratory analysis into the oral microbiome of AUD and gives a comprehensive view of oral microbiome changes after sampling 10 times longitudinally during alcohol abstinence after sustained heavy alcohol use.

In conclusion, this study demonstrated that the oral microbiome is strongly associated with alcohol of choice in treatment seeking individuals with AUD, and dynamic changes in health and disease-associated genera occur during abstinence following heavy alcohol consumption. We observed poor oral health in individuals with AUD upon the entry into treatment and an overall improvement in oral health during abstinence. While we cannot attribute the poor oral health observed specifically to heavy drinking, we can comment on the association of it with AUD. A reduction in alpha diversity and also with reduced abundance of periodontitis-associated bacteria paralleled this change. As periodontal disease is associated with medical conditions such as cardiovascular disease, diabetes mellitus and oral cancer [5], special attention to the oral health of patients with AUD may contribute to risk reduction of comorbid chronic diseases. Bacteria of the oral microbiome identified in this research may serve as valuable prognostic biomarkers linking heavy alcohol use to poor health outcomes.

## Supplementary Material

Supplemental Material

## Data Availability

All raw sequencing data acquired in this study have been deposited to the National Center for Biotechnology Information Sequence Read Archive (NCBI/SRA) (www.ncbi.nlm.ni.gov/sra) under BioProjectID PRJNA659632. The METAGENOTE tool from the National Institute of Allergy and Infectious Diseases (NIAID) Office of Cyber Infrastructure and Computational Biology (OCICB) in Bethesda, MD was used to facilitate data upload [58]. Reconstructed counts for the entire dataset are available in Supplemental Table 1.
