# Supplementary material for: The oral microbiome in alcohol use disorder: a longitudinal analysis during inpatient treatment
Source: J Oral Microbiol. 2021 Dec 1;14(1):2004790. doi: 10.1080/20002297.2021.2004790 (PMC8648028; doi:10.1080/20002297.2021.2004790)
Supplement: Supplemental Material [file ZJOM_A_2004790_SM5851.zip › Supplementray files/Supplemental_Materials_Redo_Oct2021_clean.docx]

**Supplemental Materials Documentation:
*The Oral Microbiome in Alcohol Use Disorder: A longitudinal analysis during inpatient treatment***

***_________________________________________________________________***

**Supplemental Table 1.** Reconstructed Count Table (See Excel Document)

**Supplemental Table 2.** Longitudinal Relative Abundance Comparisons for all genera *(See Excel Document)*

**Supplemental Table 3**. Periodontitis-Associated Genera Comparisons between periodontal disease groups

|  | Mean Relative Abundance (%) | | | | ***Prob>\|Z\|*** | |
| --- | --- | --- | --- | --- | --- | --- |
|  | Day 1-2 | | Week 4-5 | |  |  |
| **Genus** | **N/M** | **M/S** | **N/M** | **M/S** | **Day 1-2** | **Week3-4** |
| *Campylobacter* | 0.92 | 1 | 0.64 | 0.82 | *0.97* | *0.62* |
| *Catonella* | 0.08 | 0.07 | 0.11 | 0.05 | *0.34* | *0.04* |
| *Desulfobulbus* | 0 | 0.03 | 0 | 0.01 | *0.09* | *0.23* |
| *Dialister* | 0.02 | 0.13 | 0.01 | 0.05 | *0.03* | *0.01* |
| *Filifactor* | 0.01 | 0.18 | 0.01 | 0.07 | *0.05* | *0.28* |
| *Lactobacillus* | 0.05 | 1.35 | 1.03 | 0.12 | *0.04* | *0.94* |
| *Megasphaera* | 0.39 | 0.51 | 0.2 | 0.34 | *0.85* | *0.09* |
| *Peptostreptococcus* | 0.39 | 0.19 | 0.23 | 0.14 | *0.34* | *0.18* |
| *Porphyromonas* | 2.26 | 1.37 | 3.56 | 1.22 | *0.31* | *0.04* |
| *Selenomonas* | 0.05 | 0.08 | 0.02 | 0.08 | *0.22* | *0.14* |
| *Treponema* | 0.01 | 0.15 | 0.06 | 0.07 | *0.02* | *0.64* |

**Supplemental Figure 1.** Oral specimen collection chart through treatment program for Alcohol Use Disorder


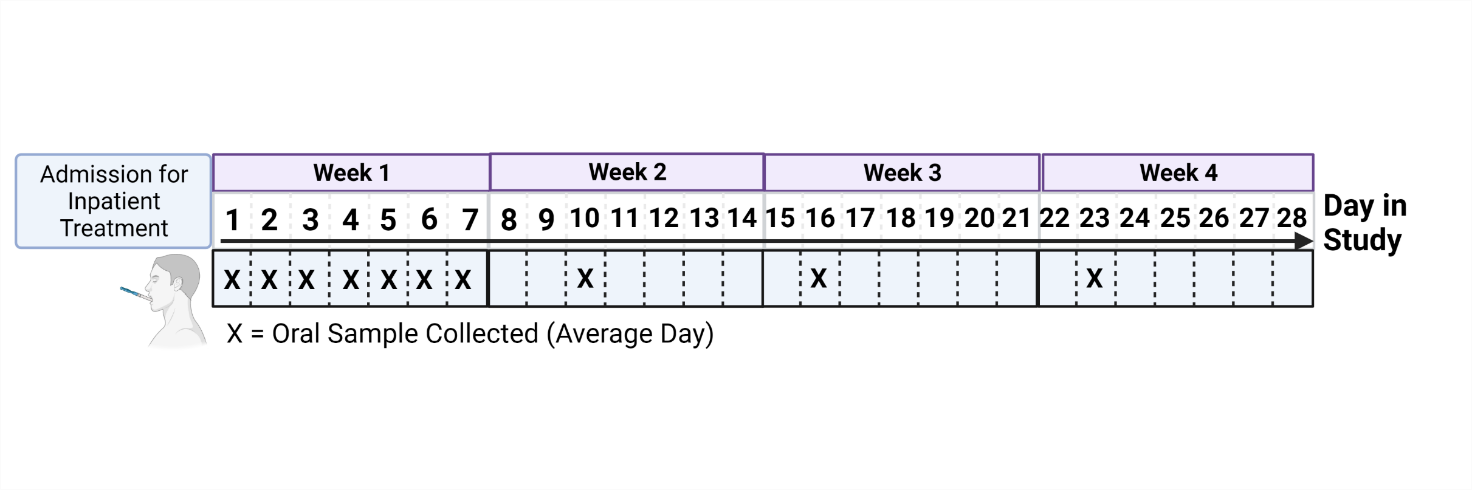


Oral sampling collection diagram showing an oral sample taken daily for the first week and then once per week during the remaining three weeks of inpatient treatment. Sampling at weeks 2-4 did not always occur on the same day for each patient.

**Supplemental Figure 2.** **Oral health in Alcohol Use Disorder**


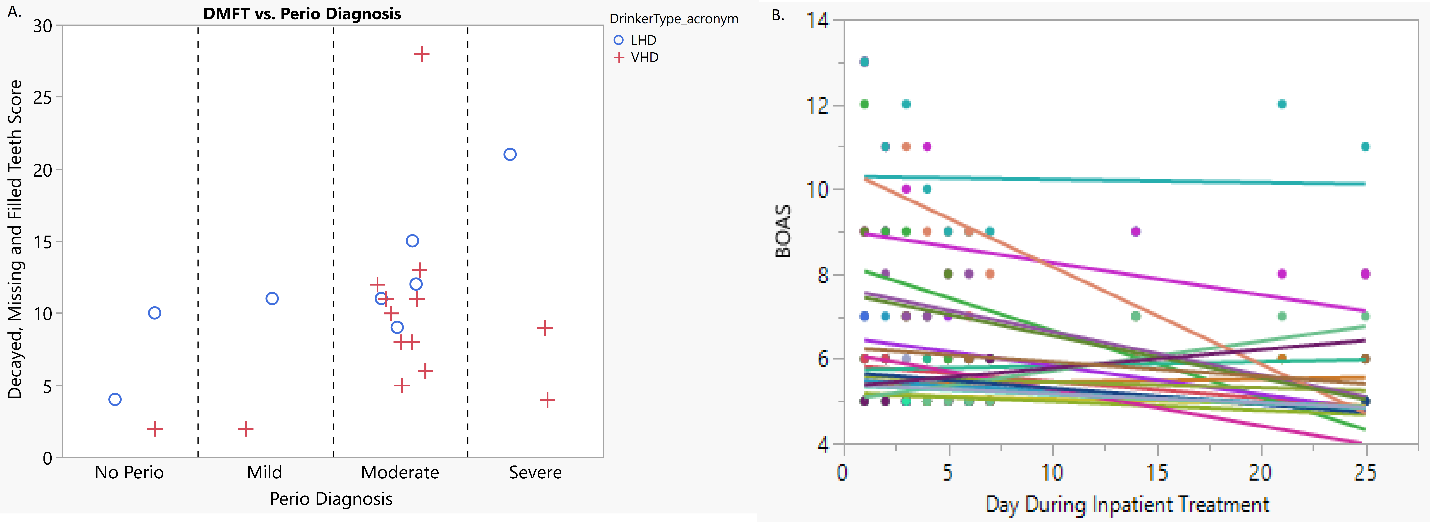


**2A**) DMFT score (y-axis) stratified by periodontal disease diagnoses (x-axis) by drinking consumption groups, VHD (red crosses) and LHD (blue circles). Each point on the plot represents an individual patient’s DMFT value at the start of treatment. No significant difference in DMFT values found between periodontal disease groups. **2B**) Beck’s Oral Assessment Score (y-axis) recorded at each sampling day during treatment (x-axis). Each dot and color represent an individual patient. Each line represents an individual patient’s line of fit through treatment (F: 4.74, *P<* 0.04).

**Supplemental Figure 3.** **Shannon Diversity Analysis by variables of interest**

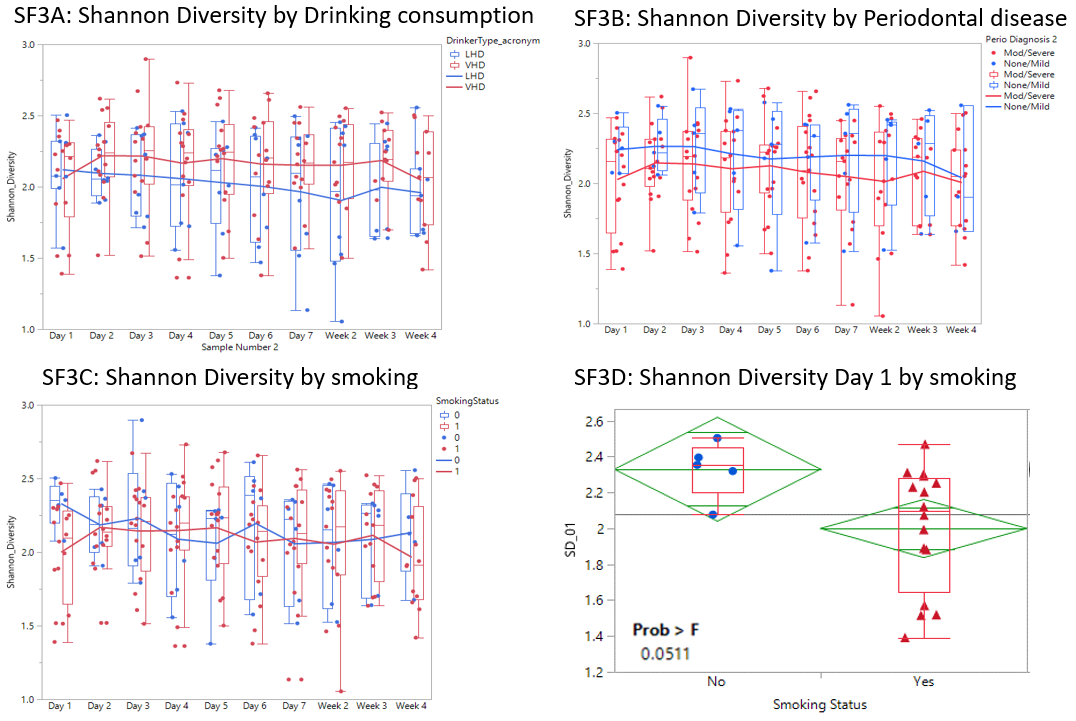


Shannon Diversity index (y-axis) at each sampling time point (x-axis) by: (**3A**) drinking consumption amount. VHD (red) shows lower average SDI during treatment than LHD (blue). **3B**) periodontal disease group (N/M: blue, M/S: red). **3C**) smoking (smoking: red, non-smoking: blue). **3D**) Shannon Diversity index (y-axis) at day 1 by smoking status (x-axis). Two group t-test comparing average SDI found to be not significant (*P =* 0.051). Abbreviations: VHD (very heavy drinkers; i.e. ≥ 10 drinks/day); LHD (less heavy drinkers; i.e. < 10 drinks/day); N/M: None and mild (periodontal disease status); Moderate and severe (periodontal disease status); SDI: Shannon diversity index.

**Supplemental Figure 4. Total number of nenera by alcohol choice group**

**
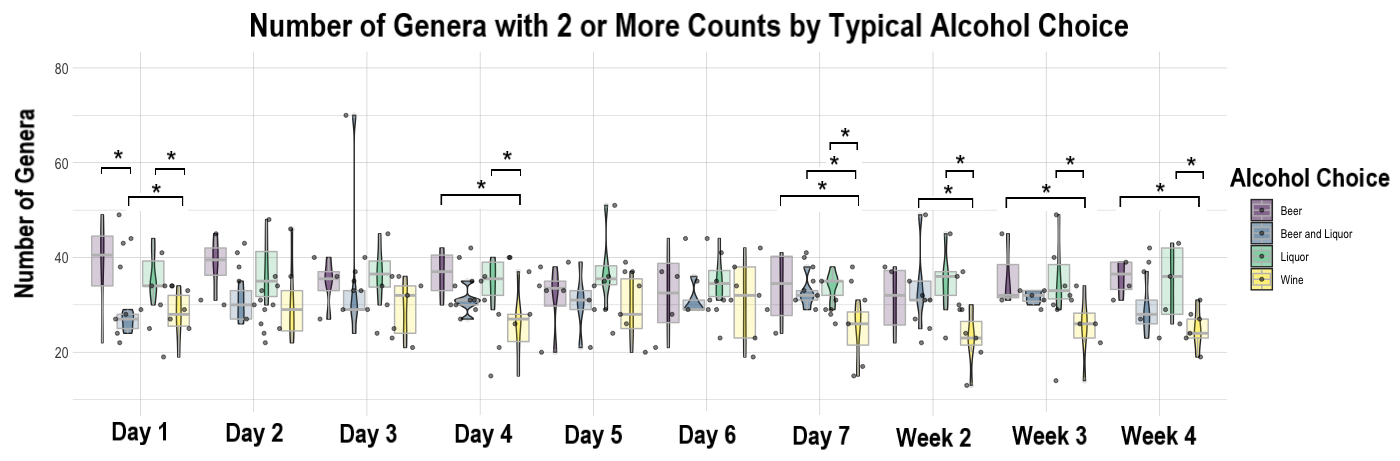
**

Total number of genera within each sample across treatment program stratified by alcohol preference. Significant difference between alcohol preference type found at days 1, 4 and 7 and weeks 2 through 4.

**Supplemental Figure 5. PCA longitudinal pairwise sample comparison between day 2 and all other sampling time points**


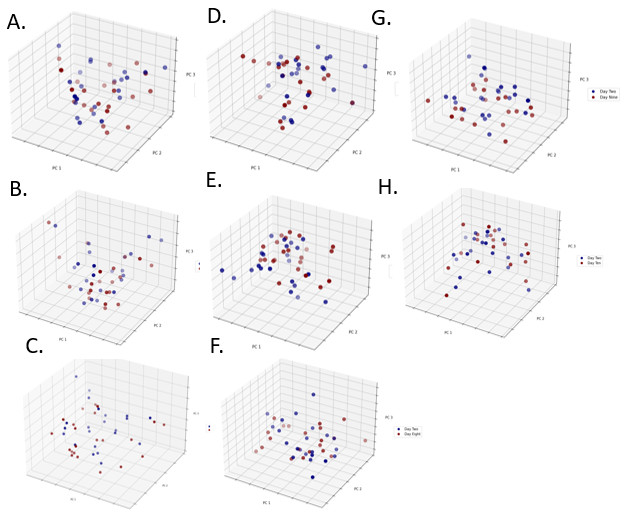


**5A-H**. Principal Components Analysis (PCA) plots of oral microbiome samples, performed at the taxonomic level of genus and based on Bray-Curtis dissimilarity stratified by longitudinal samples by day two compared to day three (ANOSIM based on Bray-Curtis dissimilarity; **A**. R = -0.033, P = 0.914), **B.** day four (R = -0.014, *P* = 0.639), **C**. day 5 (R = 0.002, *P* = 0.378), **D**. day six (R = 0.004, *P* = 0.391), **E**. day seven (R = 0.003, *P* = 0.39), **G**. week 2 (R = 0.010, *P* = 0.284), **G**. week 3 (R = -0.027, *P* = 0.793) or **H**. week 4 (R = -0.014, *P* = 0.6).

**Supplemental Figure 6. Beta Diversity differences between Less Heavy Drinking and Very Heavy Drinking groups**
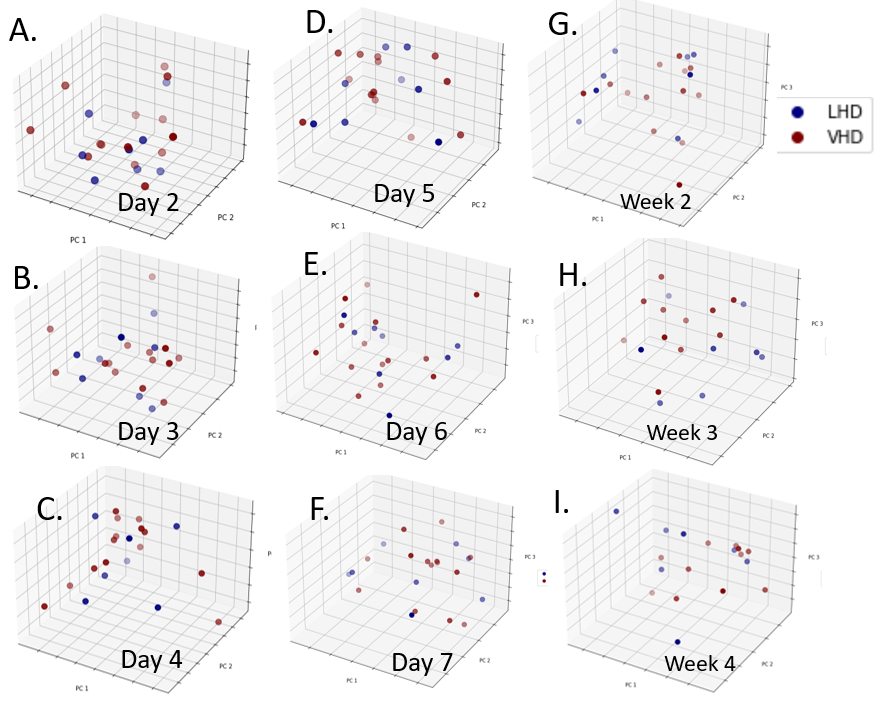


**6A–I**. Principal Components Analysis (PCA) plots of oral microbiome samples, performed at the taxonomic level of genus and based on Bray-Curtis dissimilarity stratified by Less Heavy Drinking (LHD) and Very Heavy Drinking (VHD) groups. Overall gut microbial community structure was not significantly different between VHD and LHD groups, as determined by analysis of similarity (ANOSIM) analysis, at **A**. sample two (R = -0.142, P = 0.984), **B**: sample three (R = -0.115, P = 0.948), **C**: sample four (R = -0.067, P = 0.716), **D**: sample five (R = -0.024, P = 0.539), **E**: sample six (R = -0.037, P = 0.63), **F**: sample seven (R = 0.089, P = 0.118), **G**: sample eight (R = 0.006, P = 0.398), **H**: sample nine (R = 0.059, P = 0.198), **I**: sample ten (R = 0.007, P = 0.422).

**Supplemental Figure 7. Beta Diversity differences between smoking status groups**


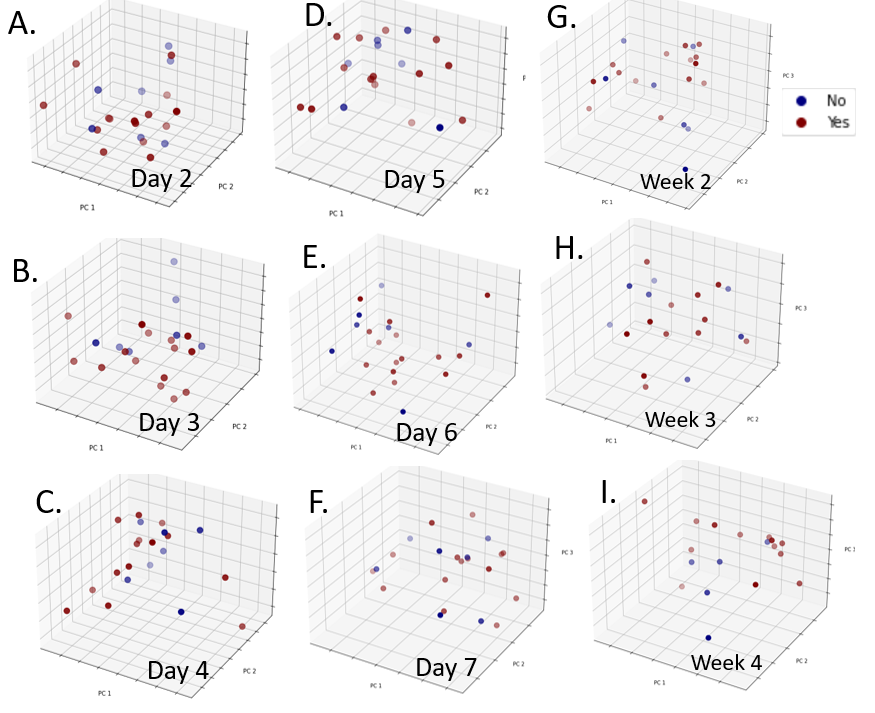


**7A–I.** Principal Components Analysis (PCA) plots of oral microbiome samples, performed at the taxonomic level of genus and based on Bray-Curtis dissimilarity stratified by smoking status (yes/no) groups. Overall gut microbial community structure was not significantly different between smoking groups (with the exception of sample nine), as determined by analysis of similarity (ANOSIM) analysis, at **A**. sample two (R = 0.036, *P* = 0.356), **B**: sample three (R = 0.118, *P* = 0.132), **C**: sample four (R = 0.038, *P* = 0.283), **D**: sample five (R = -0.032, *P* = 0.559), **E**: sample six (R = 0.101, *P* = 0.15), **F**: sample seven (R = 0.034, *P* = 0.304), **G**: sample eight (R = -0.009, *P* = 0.462), **H**: sample nine (R = 0.223, *P* = 0.021), **I**: sample ten (R = -0.017, *P* = 0.501).

**Supplemental Figure 8. Beta Diversity differences between periodontal disease status groups**


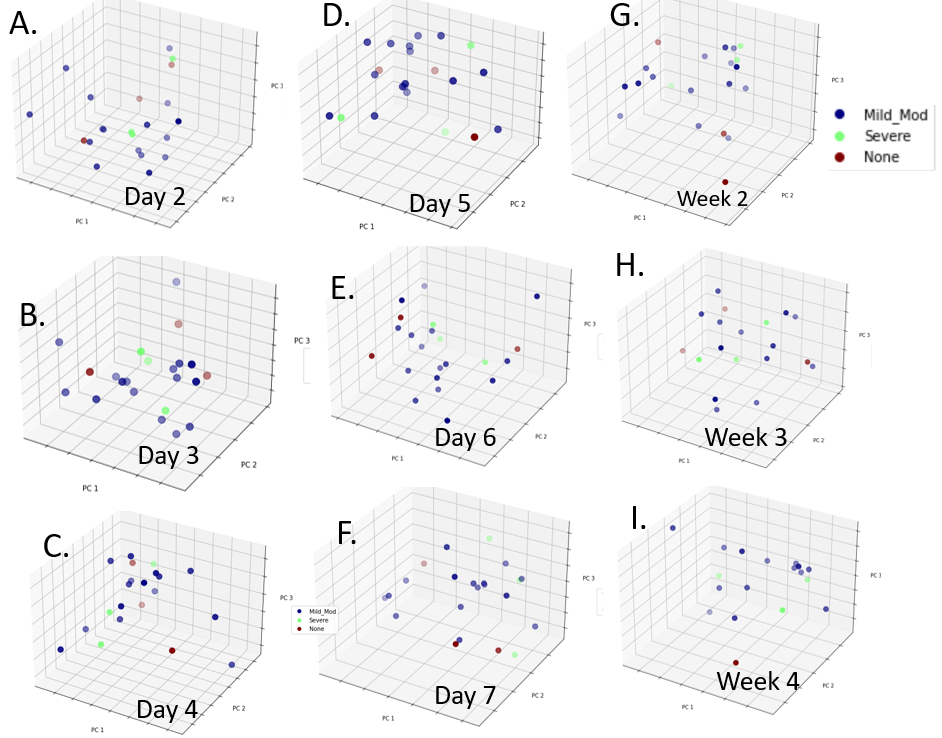


**8A–I.** Principal Components Analysis (PCA) plots of oral microbiome samples, performed at the taxonomic level of genus and based on Bray-Curtis dissimilarity stratified by periodontal disease status (none/mild [N/M] versus moderate/severe [M/S]) groups. Overall gut microbial community structure was not significantly different between periodontal disease groups, as determined by analysis of similarity (ANOSIM) analysis, at **A**: sample two (R = -0.120, *P* = 0.806), **B**: sample three (R = -0.057, *P* = 0.629), **C**: sample four (R = -0.063, *P* = 0.667), **D**: sample five (R = -0.019, *P* = 0.552), **E**: sample six (R = -0.083, *P* = 0.714), **F**: sample seven (R = 0.092, *P* = 0.193), **G**: sample eight (R = 0.211, *P* = 0.078), **H**: sample nine (R = 0.189, *P* = 0.082), **I**: sample ten (R = 0.283, *P* = 0.061).

**Supplemental Figure 9**. Patient relative abundance plots and Shannon Diversity changes through treatment. Plotted in SDI range descending order.


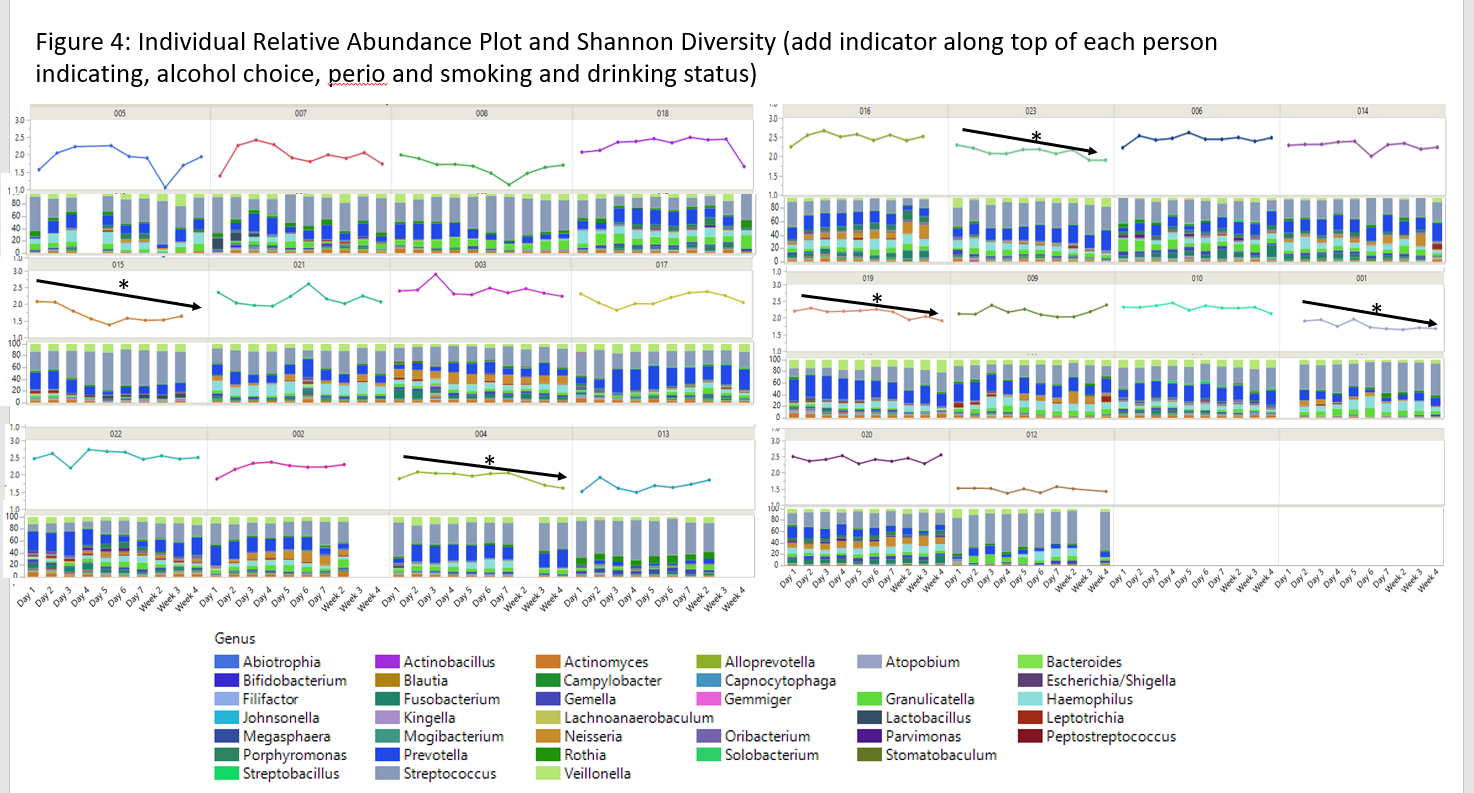


Individual patient relative abundance plots over the ten sampling time points during inpatient treatment for AUD. The within patient SDI range (maximum SDI over ten time points minus the minimum SDI over ten time points) was calculated for each patient and relative abundance plots were created. The individual relative abundance plots are organized in descending order of SDI range over time, i.e. patients with the largest SDI spread across the ten time points (largest SDI range: 1.2) are plotted first and each patient is plotted in descending order down to the patient with the lowest SDI spread (lowest SDI spread: 0.23). Patient specific SDI changes were assessed using linear regression across the time points to identify individual based diversity changes during alcohol cessation. * *P* < 0.05 for significantly decreasing SDI over time. Five patients exhibited significant decreased longitudinal SDI change denoted by * and arrow above RA plot. Abbreviations: SDI Shannon diversity index

**Supplemental Figure 10.** Venn Diagram Overlap and Log Relative Abundance differences of all genera changes between: Day 7/ Day 2, Week 2/ Day 2, Week 3/Day 2, Week 4/Day 2


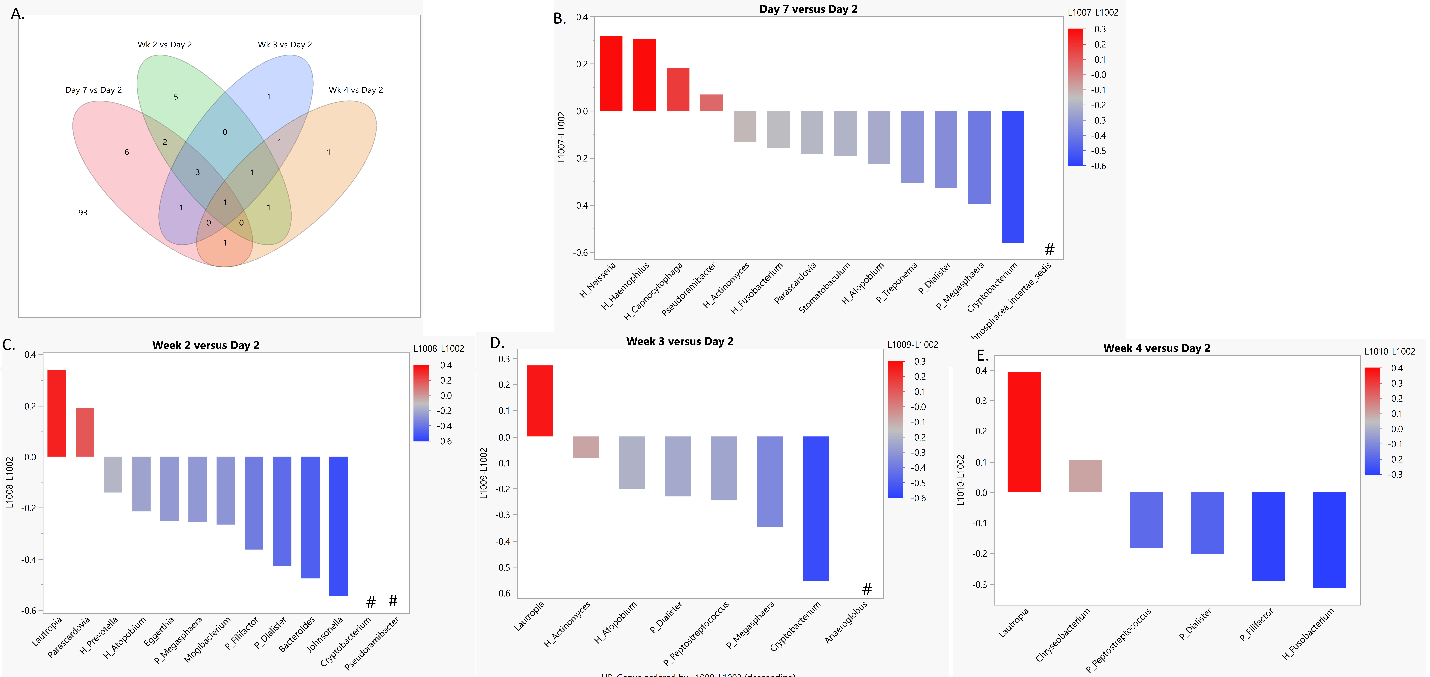

Venn Diagram overlap **(A**) of genera found to be significantly changing between Day 7- Day 2 **(B)**, Week 2-Day 2 **(C)**, Week 3-Day 2 **(D)**, Week 4-Day 2 **(E)**. Red blue bar charts show log 10 relative abundance change between the four comparisons of interest. All genera shown are significantly different between comparison of interest (**P* < 0.05). (#Note: Relative abundance change plots missing genera with 0 counts for one of the time points as cannot take log transform of 0. The following genera are missing; Day 7- Day 2: *Lachnospiracea_incertae_sedis*, Week 8 – Day 2: *Cryptobacterium*, *Pseudoramibacter*; Week 9 – Day 2: *Anaeroglobus*)

**Supplemental Figure 11.** Average relative abundance of health- and periodontal disease-associated genera in HMP vs AUD subjects at Day 2 and Week 4.


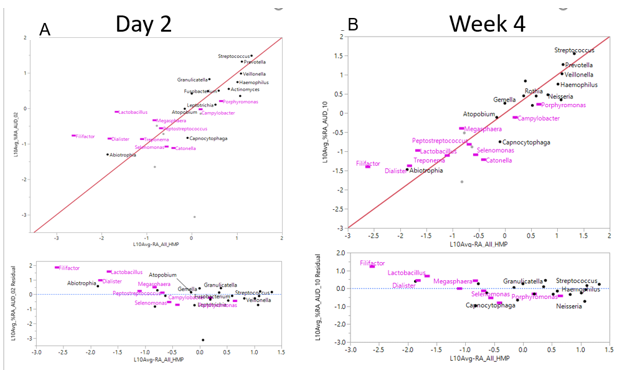


Figure Legend: Average Abundance and line of identity residuals between HMP and AUD at day 2 **(A)** and at week 4 **(B)**. Pink rectangles refer to ‘periodontitis-associated’ genera and black dots refer to ‘health-associated’ genera. **A)** Correlation between average abundance of HMP (x-axis) and average abundance of AUD at day 2 (y-axis). Lower panel shows day 2 AUD residuals (y-axis) from line of identity fit with HMP. **B)** Correlation between average abundance of HMP (x-axis) and average abundance of AUD at week 4 (y-axis). Lower panel shows week 4 AUD residuals (y-axis) from line of identity fit with HMP. Abbreviations: HMP Human Microbiome Project; AUD Alcohol Use Disorder

**Supplemental Figure 12.** Comparison of periodontitis-associated genera on tongue dorsum in patients with AUD diagnosed with Severe/Moderate vs None/Mild periodontal disease


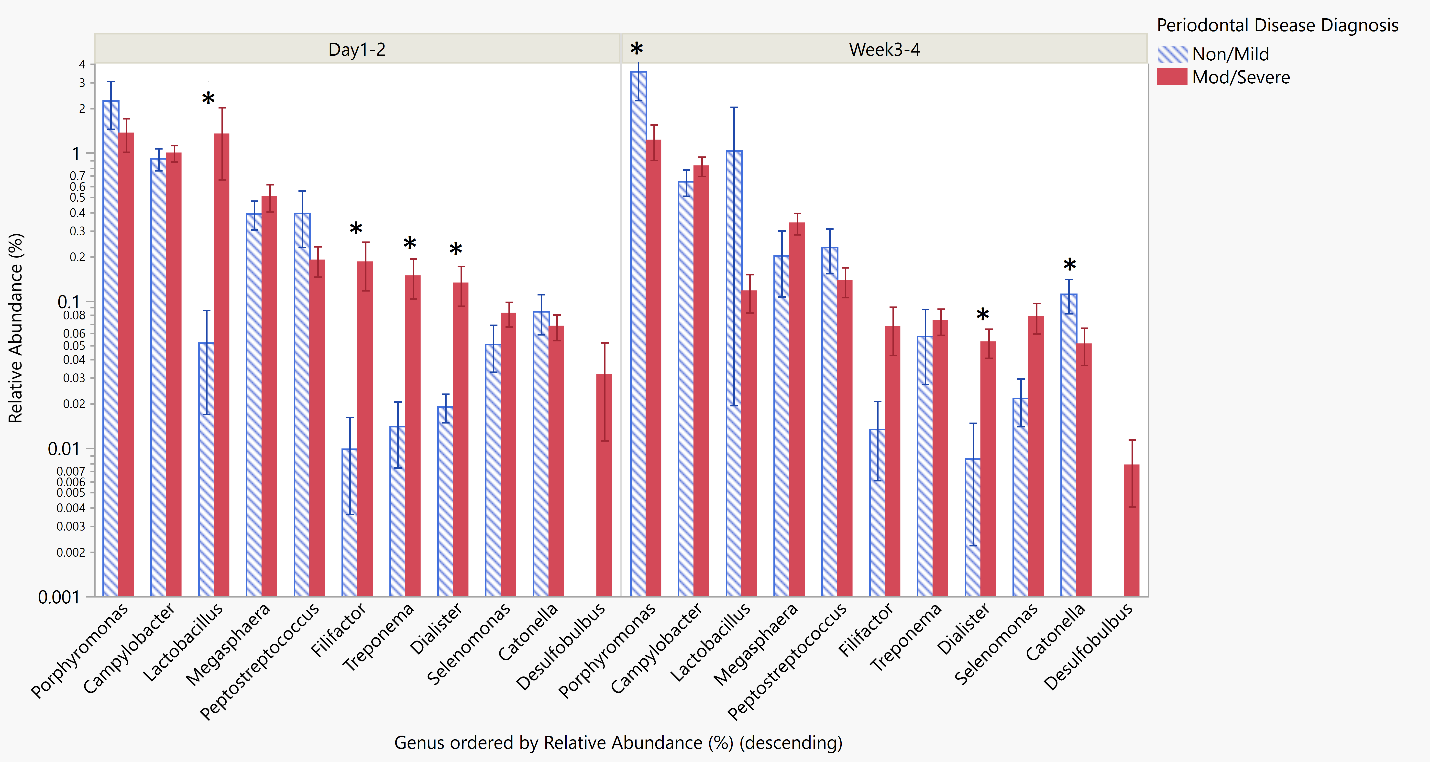


Average Abundance (y-axis) of periodontitis-associated genera (x-axis) in none/mild (blue striped) versus moderate/severe (red solid) periodontal disease diagnosis groups at start of treatment **A)**, average of days 1 and 2) and last two weeks of treatment **B)**, average of weeks 3 and 4). Significant genera between periodontal disease groups denoted by * (*P* < 0.05).
