# Supplementary material for: The oral microbiome in alcohol use disorder: a longitudinal analysis during inpatient treatment
Source: J Oral Microbiol. 2021 Dec 1;14(1):2004790. doi: 10.1080/20002297.2021.2004790 (PMC8648028; doi:10.1080/20002297.2021.2004790)
Supplement: Supplemental Material [file ZJOM_A_2004790_SM5851.zip › Supplementray files/SupportingInformation_SupplementalMethods_clean.docx]

**SUPPLEMENTAL METHODS**

***Sequence processing***

Bacterial DNA sequences were mapped with Ion Torrent Suite Version 5.0.5. Raw sequence and quality data were exported from the Ion Torrent Suite using a file exporter plug-in version 5.0.3.1. Data were preprocessed using a method described previously [24].v

Briefly, reads were filtered using fastq_filter python script from USEARCH v11 (arguments fastq_max 50 and fastq_trunclen 200). An awk script was used to edit read labels on demultiplexed fasta files so that the sample identifier was retained in each read id name. Edited files were concatenated into one fasta file for further processing. Reads were split into 12 sets of data based on 6 forward and 6 reverse primer sequences (V2F, V2R, V3F, V3R, V4F, V4R, V67F, V67R, V8F, V8R, V9F and V9R) using a python script Split_on_Primer.py (download available at https://github.com/Y-Lammers/Split_on_Primer). Primer sequences were removed from each set of targeted amplicons using fastx_truncate from the fastx_toolkit suite of processing tools (http://hannonlab.cshl.edu/fastx_toolkit/). Reverse reads were reverse complemented using fastx_reverse_complement from fastx_toolkit and were subsequently concatenated to the forward reads from the same targeted V region.

In the USEARCH pipeline for zero-radius operational taxonomic units (ZOTUs) generation, singleton ZOTUs were removed from filtered and trimmed sequences using default parameters in the fastx_uniques command, denoised reads were generated using UNIOISE3, and a ZOTU table for each V region was created using the otutab command

Data were rarified to 5000 reads per sample using otutab_norm. Taxonomy was assigned using sintax and the RDP reference database version 16 (https://www.drive5.com/usearch/manual/faq_tax_db.html). Both ZOTU and taxonomy tables for each V region were exported and imported into the JMP™ Data Discovery software (SAS Headquarters, Cary, NC) for data merging and manipulation.

Once a ZOTU table was generated for each of the six hypervariable 16S regions, ZOTU’s were summarized at the genus level by summing ZOTU’s mapped to the same genus for each sample. We excluded the ZOTU table from region V9 from this analysis based on our previous finding that the V9 region has low annotation accuracy with Ion Torrent sequencing data

ZOTU counts mapped to the same genus were summed over for each genus. Each genus level table for each V region were merged by genus using our previously introduced data combination method. Count data for each V region were combined into ‘reconstructed counts’ using the root mean square where x_1_ through x_5_ refers to summed genus counts for V2, V3, V4, V67 and V8 respectively:

$RMS=\sqrt{\frac{{x_{1}}^{2}+{x_{2}}^{2}{x_{3}}^{2}{x_{4}}^{2}{x_{5}}^{2}}{5}}$

Reconstructed counts were converted to relative abundances (RA) at the genus level for all V regions. RA values from a subset of each individual’s samples (days 1-2 and weeks 2-4) were submitted to Principal Component Analysis (PCA) and PCs 1-3 were plotted in two bivariate plots. The range across each individual’s PC was calculated to investigate the intra-individual variability. Individuals with larger PC range values indicate more variability across the treatment period and individuals with smaller PC range values indicate less variability. The range over PCs 1-3 for each individual was plotted in descending order to visualize the intra-individual PC variability.

***Statistical Analyses***

Statistical analyses were performed using JMP™ version 14 Statistical Discovery Software (SAS Headquarters, Cary, NC). A one-way ANOVA was used to compare DMFT values (response variable) across the drinking consumption variable (VHD/LHD) and the two-group periodontal disease diagnosis (N/M and M/S) across the entire cohort. BOAS values were investigated across the entire patient cohort across the 10 sampling time points to assess whether oral health improved during treatment for AUD. BOAS values were also assessed over time after converting the sampling time point to a numerical value corresponding to the average day of sample collection during treatment. A linear fixed effect model was used with the total BOAS score as the response variable and the time of collection as the within subject value.

Composition of individual oral samples was evaluated by the Shannon diversity index (SDI) and the total number of genera to measure richness and evenness of individual bacterial communities. The total number of genera in each sample was calculated by summing up the total genera with a count of 2 or greater. Average SDI values were compared across the ten sampling time points to determine if there was a difference across all or between any pair of sampling time points. To investigate global linear SDI change over time, the sampling time point was recoded to a numerical value based on the day the sample was collected during the inpatient treatment. A linear fixed effect model was applied using the SDI as the response variable and the recoded numerical time variable as the within subject factor. To assess average SDI changes between any pair of sampling time points, a matched pairs t-test was conducted. To assess SDI changes between any of the four factors of interest over time, we used a mixed model analysis, using one of the four factors of interest as a fixed effect and the sampling time point as the repeated measure. The four factors tested were as follows: 1) drinking consumption (VHD, LHD), 2) alcohol type (wine, beer, beer & liquor, liquor), 3) smoking status (yes, no) and 4) periodontal disease status (N/M, M/S). Post hoc testing was carried out at each sampling time point to assess whether there were any significant differences in SDI across the four factors of interest using a one-way ANOVA or t-test. The number of genera within each sample at different time points was evaluated across the four factors of interest using a one-way ANOVA.

Binary Sorenson-Dice Dissimilarity (BSDD) Index was used to assess the microbiota dissimilarity between each patient’s baseline sample (first sample collected) compared to each successive sample thereafter, (days 2-7 and weeks 2-4) thus, up to 9 samples during treatment.

***Genera comparison between Tongue Dorsum Samples from the Human Microbiome Project and Individuals with AUD***

Tongue dorsum samples from individuals with AUD were compared to tongue dorsum samples from healthy individuals from the Human Microbiome Project (HMP) [13]. The aim of this comparison is to investigate how genera found to be highly abundant from tongue dorsum samples in health individuals compare to genera found from individuals with Alcohol Use Disorder. This was an exploratory analysis used to gain a broader understanding if highly abundant genera are affected by chronic alcohol use. To facilitate this comparison, taxonomy tables quantified as operational taxonomic unit (OTU) counts were downloaded from the Human Microbiome Project (HMP) website (HMPdacc web portal: https://www.hmpdacc.org/hmp/HMQCP/). The OTU taxonomy tables from V regions 1-3 and V regions 3-5 (Total number of Primary Sample Numbers = 320) were combined by first summing over OTUs mapped to the same genus and then by computing the root mean square across tables from the same sample, as previously detailed. We calculated the average RA across all samples in the combined HMP dataset. Any genera with at least a 0.10% average RA, corresponding to the top 20^th^ percentile in the HMP dataset, were identified as genera that are highly abundant in the oral microbiome, sampled by the tongue dorsum, of healthy individuals.

The average RA of the highly abundant genera in the HMP were compared to the average RA of those same genera in AUD using Pearson correlation. The level of agreement for each genus was assessed using the residuals generated from the line of identity. Any residual with a 0 indicates perfect agreement between the two genera compared and residuals with increased absolute values indicates increasing discordance between the genera.

***Characterization of ‘health-associated’ and ‘periodontitis-associated’ genera***

Genera previously reported by Wilbert et. al. to be abundant on the tongue dorsum of healthy individuals was investigated in this patient cohort with AUD [35]. To quantify bacterial taxa (genus-level) that are specific to the healthy human tongue dorsum, we used a two-part validation: 1) taxonomy tables from the HMP were used to identify taxa on healthy tongue dorsum samples and 2) taxa from tongue dorsum samples identified in previously published research. Genera found on both validation sets were categorized as ‘health-associated’. These genera include *Abiotrophia, Actinomyces, Atopobium, Capnocytophaga, Fusobacterium, Gemella, Granulicatella, Haemophilus, Leptotrichia, Neisseria, Prevotella, Rothia, Streptococcus and Veillonella.*

Additionally, genera found in shallow and deep sites of plaque samples in periodontal disease from previous research, deemed ‘periodontitis-associated’, were investigated in this cohort of individuals with AUD [36, 37]. The “periodontitis-associated” genera investigated include: *Peptostreptococcus, Filifactor, Porphyromonas, Lactobacillus, Megasphaera, Desulfobulbus, Campylobacter, Selenomonas*, *Dialister, Treponema,* and *Catonella*.

***Longitudinal relative abundance changes of genera during treatment for AUD***

The longitudinal relative abundance change for each genus was investigated between multiple time points during treatment. Each genus in the dataset was tested using a Wilcoxon signed-rank test for paired data between multiple time point comparisons as follows: day 2 versus day 7, day 2 versus week 2, day 2 versus week 3 and day 2 versus week 4. To visualize the change between the two time points, average RA values were transformed using a logarithm base 10 and the difference between the log 10 RAs between day 2 and week 2, week 3 and week 4, respectively, was calculated. Subsequently, the log RA difference of the health- or periodontitis-associated genera were grouped and plotted.

The average RA of the combined ‘health-‘ and ‘periodontitis-‘ associated genera in the HMP were plotted against the same genera in our cohort at day 2 and again at week 4. We determined overall agreement between the RA of ‘health-‘ and ‘periodontitis-‘ associated genera in HMP and AUD individuals by Pearson correlations at day 2 and week 4. Agreement between the average RA for specific genera between HMP and AUD individuals was determined by residual from the correlation line with 0.0 indicating perfect agreement and higher numbers (absolute value) indicating less agreement. Negative residuals are associated with higher mean RA of the particular genus in the HMP cohort, while positive residuals are associated with high mean RA in the AUD cohort.

To assess differences between the periodontal disease groups (N/M versus M/S) in this patient cohort and in the periodontitis-associated genera, the two periodontal disease groups were compared in the baseline samples (average of days 1 and 2 samples) and in the end of treatment samples (average of the weeks 3 and 4 samples) using a Wilcoxon signed-rank test. We plotted average abundances at baseline and at the end of treatment using a logarithm base 10 transform. The difference between the logarithm base 10 relative abundance between N/M versus M/S groups were shown for each genus.
